# Effects of the single and combined effect of music and other strategies on combat sport performance: a systematic review and meta-analysis

**DOI:** 10.3389/fspor.2026.1733470

**Published:** 2026-03-02

**Authors:** Nidhal Jebabli, Wissem Dhahbi, Manar Boujabli, Mariem Khlifi, Nejmeddine Ouerghi, Anissa Bouassida, Abderraouf Ben Abderrahman, Roland van den Tillaar

**Affiliations:** 1 Research Unit: Sport Sciences, Health and Movement, High Institute of Sport and Physical Education of Kef, University of Jendouba, Kef, Tunisia; 2 European Institute for Talents, Education, Research & Development, Split, Croatia; 3 Qatar Police Academy, Police College, Training Department, Doha, Qatar; 4 Higher Institute of Sport and Physical Education of Ksar-Said, University of Manouba, Manouba, Tunisia; 5 Department of Sports Science and Physical Education, Nord University, Levanger, Norway

**Keywords:** auditory stimulation, ergogenic aids, exercise martial arts, physical performance, psychophysiological responses

## Abstract

**Introduction:**

There is a lack of systematic mechanism regarding the single and combined effect of listening to music with other strategies on the physical and psychophysiological performance of combat sport athletes. This systematic review and meta-analysis examined the single and combined effects of musical interventions on the technical, physical, physiological, and psychological performance of combat sports athletes, while identifying possible synergistic ergogenic strategies with music.

**Methods:**

A systematic search was conducted across five electronic databases (PubMed/MEDLINE, Web of Science, Scopus, SPORTDiscus, and ScienceDirect) following PRISMA guidelines. Methodological quality was assessed using the PEDro scale. We performed a meta-analysis addressing physical, physiological and psychological function.

**Results:**

Fifteen studies met the inclusion criteria, with a total of 1,456 participants. Music-only interventions demonstrated a small beneficial effect (*d* = 0.19, 95% CI: 0.02–0.36, *p* = 0.021). Subgroup analyses revealed psychological outcomes showed the strongest response (*d* = 0.52, *p* = 0.011), while physical performance effects were variable (*d* = 0.18, *p* = 0.583) and physiological measures showed minimal impact (*d* = 0.05, *p* = 0.921). Combined interventions demonstrated substantially larger effects than music alone (*d* = 0.93 vs. *d* = 0.19), with music and caffeine showing the greatest synergistic benefit (*d* = 1.24).

**Conclusion:**

Music interventions alone produce small beneficial effects on combat sport performance, with strongest impacts on psychological outcomes. However, combined interventions demonstrate superior efficacy, particularly music and caffeine supplementation, suggesting multimodal approaches optimize performance enhancement in combat sports.

**Systematic Review Registration:**

https://www.crd.york.ac.uk/PROSPERO/view/CRD420251073337, identifier CRD420251073337.

## 1 Introduction

Striking combat sports, encompassing kickboxing, taekwondo, and karate, represent a unique category of athletic endeavors characterized by high-intensity, primarily powered by oxidative energy systems with a specific anaerobic pathway for scoring actions that require the simultaneous integration of advanced technical, tactical, physical, and psychological skills (1–3). These disciplines present athletes with alternating sequences of explosive efforts and brief recovery periods under conditions of accumulating fatigue, demanding comprehensive preparation that extends beyond traditional physical conditioning to encompass power development, reaction speed optimization, motor coordination refinement, and resilience to psychophysiological stress (4, 5). This multifaceted performance profile necessitates a holistic approach to performance enhancement that addresses biomechanical, cognitive, and emotional components within an integrated training framework. In contemporary competitive environments, strength and conditioning professionals increasingly seek ergogenic interventions that can enhance performance while maintaining compliance with anti-doping regulations. The pursuit of optimal athletic performance has evolved beyond traditional nutritional and pharmacological approaches to encompass psychological and psychophysiological interventions that operate through distinct neurobiological pathways (6). Among these emerging modalities, music has gained recognition as an accessible, adaptable, and scientifically validated ergogenic aid that influences multiple performance-relevant systems simultaneously (7).

Understanding music's potential as a performance-enhancing intervention requires examining the complex neurobiological processes through which auditory stimuli influence human behavior and athletic performance. Music listening activates the brain's reward system through dopaminergic pathways in the mesolimbic circuit, creating neurochemical responses analogous to those observed with primary biological rewards (8). This activation occurs through a sophisticated temporal dissociation whereby the caudate nucleus becomes active during anticipation of musical pleasure, while the nucleus accumbens responds during the actual experience of rewarding musical moments, resulting in measurable dopamine release that influences motivation, mood regulation, and stress perception (9). The ergogenic effects of music extend beyond simple psychological enhancement to encompass measurable physiological adaptations that directly impact performance capacity. Recent investigations have demonstrated that music interventions reduce resting heart rate and enhance parasympathetic recovery during rest periods (10, 11), while simultaneously increasing catecholamine levels that facilitate energy mobilization (12). These effects, enhanced recovery combined with optimized arousal, create an ideal physiological state for high-intensity intermittent activities characteristic of combat sports. Music also modulates cortical arousal and perceived exertion through mechanisms involving the regulation of perceived neural fatigue (13). A comprehensive meta-analytic review by Terry et al. (14) analyzing 139 studies with 3,912 participants demonstrated that music interventions produce small-to-moderate beneficial effects on exercise performance, with effect sizes ranging from 0.31 for endurance tasks to 0.42 for high-intensity activities. These effects appear to operate through multiple concurrent mechanisms including dissociative attention, movement synchronization with musical rhythm, and arousal regulation optimized for specific task demands.

Recent investigations have provided compelling evidence for music's ergogenic potential specifically within combat sports contexts. Studies examining self-selected music interventions in striking-based disciplines have demonstrated strongly stimulating effects on intrinsic motivation while simultaneously reducing perceived exertion and increasing pleasure sensations (11, 15, 16). These psychological adaptations translate into measurable performance improvements including enhanced reaction time, faster decision-making in competitive contexts, and improved technical accuracy during high-intensity efforts (10). From a motor control perspective, music interventions have been associated with increased intersegmental coordination and enhanced striking force, attributed to increased synchronization between auditory and motor brain areas (17). The rhythmic structure of music appears particularly relevant for combat sports, where optimal technique execution often requires precise timing and coordinated movement patterns. This synchronization effect extends beyond simple metronome-like pacing to encompass complex movement sequences that characterize sport-specific skills in striking disciplines. Importantly, research has identified sex-based differences in responsiveness to music interventions, with female athletes demonstrating enhanced sensitivity to musical characteristics and more pronounced mood improvements (11). These findings underscore the importance of individualized music selection based on athletes' sensory and psychological profiles, suggesting that optimal intervention protocols must account for individual variability in music perception and preference.

Perhaps most intriguingly, emerging evidence suggests that music's ergogenic effects are substantially amplified when combined with other performance enhancement strategies. Studies investigating multimodal approaches have demonstrated synergistic effects when music is paired with video feedback (17), caffeine ingestion (10, 18, 19), strategic napping (20), or specialized training protocols such as plyometrics (21). These combination studies reveal effect sizes substantially larger than those observed with music-only interventions, suggesting additive or potentially synergistic neurobiological mechanisms. For example, Delleli, Ouergui (10) demonstrated that combining music listening during warm-up with low-dose caffeine ingestion (3 mg·kg^−1^) produced superior improvements in combat activity profiles, reduced ratings of perceived exertion and heart rate responses, and enhanced psychological outcomes compared to either intervention administered independently. Also Caffeine consumption during exercise further stimulates dopamine release without inhibiting serotonin, which may help reduce central fatigue (22). This synergistic effect likely reflects the convergent influence of music's dopaminergic activation and caffeine's adenosine receptor antagonism on central nervous system arousal and motivation systems. The theoretical foundation for these synergistic effects lies in the complementary neurobiological pathways activated by different intervention modalities. While music primarily influences psychological states through reward system activation, other ergogenic aids may target peripheral physiological processes, cognitive function, or recovery mechanisms. When combined strategically, these interventions may produce additive benefits that exceed the sum of their individual effects.

Various meta-analyses (14, 23, 24) examined identified all the studies that investigated the impact of music on physical, psychological, and physiological changes without selection on either disciplinary aspect.

A systematic review of the literature would be pertinent to elucidate the effectiveness of music listening interventions as a solitary or complementary strategy with other complementary strategies on combat sports athletes' physical, physiological, and psychological variables. The review would take into account the type of musical stimuli (e.g., type of music, tempo, frequency) and time of administration of the interventions (e.g., pre-test, during test, post-test).

## 2 Methods

### 2.1 Protocol registration and reporting guidelines

This systematic review and meta-analysis was conducted according to the Preferred Reporting Items for Systematic Reviews and Meta-Analyses (PRISMA) 2020 statement guidelines (25) and its corresponding explanation and elaboration document. The review protocol was prospectively registered with PROSPERO (Registration ID: CRD420251073337; https://www.crd.york.ac.uk/PROSPERO/view/CRD420251073337), with any protocol deviations documented systematically and reported in accordance with contemporary standards for systematic review research.

### 2.2 Eligibility criteria

Study inclusion and exclusion criteria were defined according to the PICOS framework (26), ensuring comprehensive coverage while maintaining methodological rigor. The target population comprised combat sport athletes aged ≥15 years with minimum one-year structured training experience in recognized disciplines including taekwondo, kickboxing, karate, judo, wrestling, mixed martial arts, or boxing. Eligible interventions encompassed music-based performance enhancement strategies administered standalone or combined with evidence-based modalities (caffeine supplementation, video feedback, plyometric training, strategic napping, or knowledge of endpoint disclosure). Comparison conditions included no-music controls, alternative auditory stimuli, silence, or standardized protocols, with combination studies utilizing co-interventions without music components. Outcome measures were categorized into technical performance (reaction time, movement accuracy, skill execution), physical performance (power, strength, agility, endurance), physiological responses (cardiovascular, metabolic, stress markers), and psychological outcomes (motivation, mood, perceived exertion, arousal, cognition). Study designs were restricted to controlled experimental designs permitting causal inference, including randomized controlled trials, randomized crossover trials, and controlled crossover studies. Exclusion criteria encompassed non-athlete populations, insufficient combat sport experience (<1 year), observational studies, case series/reports, qualitative designs, studies without control conditions, insufficient quantitative data, populations with chronic illness or musculoskeletal injury, conference abstracts without full-text access, duplicate publications, and non-English/French language studies. Language restrictions limited inclusion to English and French publications. Non-English/French records identified during screening (*n* = 23) were excluded based solely on language eligibility.

### 2.3 Information sources and search strategy

A comprehensive search strategy was implemented across five major electronic databases selected for optimal coverage: PubMed/MEDLINE, Web of Science Core Collection, Scopus, SPORTDiscus via EBSCOhost and ScienceDirect, from the first registration until June 15, 2025. The search strategy employed Medical Subject Headings and free-text keywords across three domains: music interventions, combat sports, and performance outcomes, with Boolean operators applied for optimal sensitivity. Final searches were executed June 15, 2025. Supplementary search methodology included reference harvesting from included studies and relevant reviews, forward citation searching via Google Scholar and Web of Science, grey literature examination through specialized databases, expert consultation with prominent researchers, and trial registry searches (ClinicalTrials.gov, WHO International Clinical Trials Registry Platform) to identify unpublished completed studies.

The music intervention domain incorporated terms encompassing auditory stimulation modalities, including “music,” OR “auditory stimulation,” OR “sound,” OR “musical intervention,” “audio feedback,” OR “sonic enhancement”. Using Boolean operator “AND”, combat sport terminology encompassed both broad categorical terms and sport-specific descriptors, including “combat sport*,” OR “martial art*,” OR “fighting sport*,” OR “taekwondo,” OR “kickboxing,” “karate,” OR “boxing,” OR “mixed martial arts,” OR “MMA,”. Performance outcome descriptors included “performance,” OR “athletic performance,” OR “exercise performance,” OR “physical performance,” OR “physiological response*,” OR “psychological response*,” “motor performance,” OR “skill execution,” OR “competitive performance.”

Combination intervention terms reflected the multimodal nature of contemporary performance enhancement research, incorporating “caffeine,” OR “video feedback,” OR “knowledge of endpoint,” OR “knowledge of results,” OR “plyometric*,” OR “power training,” OR “napping,” OR “sleep,” OR “rest,” OR “recovery” combined with music-related terms using appropriate Boolean logic.

### 2.4 Study selection and screening

Study selection followed a rigorous two-stage screening process using Covidence systematic review software (Veritas Health Innovation, Melbourne, Australia), adhering to current best practices (27) Two independent reviewers conducted systematic evaluation of all retrieved records against predetermined eligibility criteria, with disagreements resolved through structured discussion and third-reviewer arbitration when consensus could not be achieved. Inter-rater agreement achieved substantial concordance (Cohen's *κ* = 0.76, 95% CI: 0.68–0.84), indicating high consistency in eligibility judgments (28). All records were managed using EndNote X21 reference management software with automated and manual duplicate detection, maintaining the PRISMA 2020 flow diagram throughout the review process.

### 2.5 Data collection and extraction

A comprehensive standardized data extraction form based on PICOS methodology underwent rigorous pilot testing on five representative studies before implementation (29). Two independent reviewers extracted data from all included studies, with discrepancies resolved through discussion or third-reviewer consultation. Extraction encompassed study characteristics (bibliographic information, design specifications, funding sources), participant characteristics (demographics, competitive level, training experience), intervention specifications (music genre, tempo, volume, duration, timing), and complete statistical data (means, standard deviations, confidence intervals, exact *p*-values, sample sizes). Missing data management incorporated systematic author contact protocols (maximum three attempts over six weeks) and established statistical estimation methods including the Hozo method for converting medians and ranges to means and standard deviations (30).

### 2.6 Quality assessment and risk of bias

Methodological quality was assessed using the validated Physiotherapy Evidence Database (31) scale (32), an 11-item instrument evaluating internal validity and statistical reporting with established reliability across diverse study populations (33). Two independent investigators conducted quality assessment with calibration exercises, resolving disagreements through structured discussion and third-reviewer adjudication when necessary. Quality scores were categorized as high (≥8 points), moderate (6–7 points), or low (≤5 points), with no studies excluded based solely on quality scores but utilized in predefined sensitivity analyses. To supplement PEDro assessment, we applied Cochrane Risk of Bias 2 (RoB 2) tool including the crossover extension (34) for all randomized crossover trials, evaluating bias arising from randomization process, period and carryover effects, deviations from intended interventions, missing outcome data, outcome measurement, and selective reporting. Risk of bias judgments (low, some concerns, high) were synthesized into an overall study-level rating. Overall evidence certainty was evaluated using the GRADE approach (35), considering study limitations, inconsistency, indirectness, imprecision, and publication bias, with potential upgrades for large effect magnitude, dose-response gradients, and residual confounding. Summary of Findings tables were constructed for key comparisons (music-only overall effect; outcome domain subgroups; music + caffeine combination) using GRADEpro software for guideline development tools GRADEpro GDT (36), with evidence certainty rated as high, moderate, low, or very low based on RoB 2 assessments, inconsistency (*I*^2^ thresholds), imprecision (confidence interval width and sample size), and publication bias.

### 2.7 Statistical analysis and synthesis methods

Standardized mean differences computed as Hedges' *g* (with small-sample bias correction) served as the primary effect size metric. For parallel-group designs, we used post-intervention means and standard deviations. For crossover trials, we computed effect sizes from paired comparisons adjusted for within-subject correlation. When correlations were unreported (69% of crossover comparisons), we assumed *r* = 0.5 consistent with meta-analytic conventions (59); sensitivity analyses (*r* = 0.3, 0.5, 0.7) confirmed robustness. Where pre-post measurements were available, we calculated change score effect sizes; design-type heterogeneity was negligible (*Q*betwee*n* = 1.83, *p* = 0.176). Positive values indicate favorable music intervention effects. Effect size interpretation followed established conventions (small *d* = 0.2, moderate *d* = 0.5, large *d* = 0.8) while considering practical significance in combat sport contexts (Cohen, 1988). Random-effects models using restricted maximum likelihood (REML) estimation were employed for all primary analyses, reflecting realistic assumptions regarding effect size variation across studies (37, 38). To address statistical dependence arising from multiple effect sizes per study and repeated-measures designs, we implemented three-level multilevel meta-analysis using the rma.mv function in metafor (39). This hierarchical structure partitions variance into sampling variance (level 1), within-study heterogeneity between effect sizes (level 2), and between-study heterogeneity (level 3), thereby accounting for non-independence without artificially inflating precision (40). Model specification included random intercepts for study identifier and effect size identifier nested within studies. For all meta-analyses, the Hartung–Knapp–Sidik–Jonkman (HKSJ) modification was implemented for confidence interval and *p*-value calculation to provide more conservative inference when assumptions are violated, particularly given *k* < 10 for most comparisons and substantial heterogeneity (*I*^2^ > 50%) (41). Between-study heterogeneity was assessed using Cochran's *Q* test (*p* < 0.10), *I*^2^ statistic (interpretive guidelines: <25% low, 25%–50% moderate, 50%–75% substantial, >75% considerable), tau-squared (*τ*^2^), *H* statistic, and prediction intervals. Predefined subgroup analyses examined combat sport type, outcome domains, intervention timing, study quality, and sample characteristics, with statistical testing using *Q*-tests for heterogeneity between groups. Meta-regression analyses explored continuous moderators when sufficient studies were available (*k* ≥ 10) using mixed-effects models. Comprehensive sensitivity analyses included leave-one-out analyses, quality-based restrictions, design-based comparisons, and statistical method variations. Specifically, we conducted sensitivity analyses varying assumed within-subject correlations for crossover trials (*r* = 0.3, 0.5, 0.7) to evaluate robustness to this parameter; pooled estimates exhibited minimal variation (Δ*d* < 0.04) with overlapping confidence intervals across all scenarios (42). Publication bias analyses were conducted for the music-only intervention comparison using study-level assessment to satisfy independence assumptions. We selected one representative effect size per study (primary outcome when pre-specified; otherwise, outcome with largest sample size), yielding *k* = 7 independent observations suitable for funnel plot construction and Egger's regression. Combined-intervention subgroups contained insufficient studies (*k* = 1–4 per combination type) for meaningful publication bias assessment. Combined interventions were stratified by temporal structure into acute within-session co-interventions (music + caffeine, music + video feedback, music + napping, music + knowledge endpoint) and chronic training-integrated co-interventions (music + plyometrics) to avoid conflating mechanistically distinct effect structures operating across different timescales. Synergistic effects were quantified as Δ*d* = *d*_combined − *d*_music-only.

### 2.8 Software and computational methods

All analyses were performed using R version 4.3.2 (R Core Team, 2023) within RStudio (RStudio Team, 2023), utilizing the metafor package (version 4.4-0) for primary meta-analytic computations (39), meta package (version 6.5-0) for alternative methods and publication bias assessment, dmetar package for advanced diagnostics, ggplot2 (version 3.4.4) for visualization, and gridExtra (version 2.3) for multi-panel graphics. Complete reproducibility is ensured through systematic code documentation using R Markdown with all materials publicly available in a permanent Open Science Framework repository (https://osf.io/tuysf/overview). The repository contains data extraction files, annotated R analysis scripts implementing three-level meta-analysis models, sensitivity analysis code, and all Supplementary Materials. Statistical significance was established at *α* = 0.05 with exact *p*-values reported, confidence intervals presented as primary uncertainty measures, and retrospective statistical power calculations performed to assess evidence base adequacy.

### 2.9 Ethical considerations and methodological transparency

This systematic review utilized exclusively published data, eliminating additional ethical approval requirements while maintaining adherence to established research synthesis standards. All data handling complied with relevant regulations and institutional guidelines, with participant confidentiality maintained through study-level data aggregation. Protocol deviations were documented systematically with transparent reporting of all analytical decisions, *post-hoc* analyses clearly distinguished from planned analyses, and regular team meetings ensuring consistent methodology application throughout the review process.

## 3 Results

### 3.1 Study selection and characteristics

**Figure 1:**
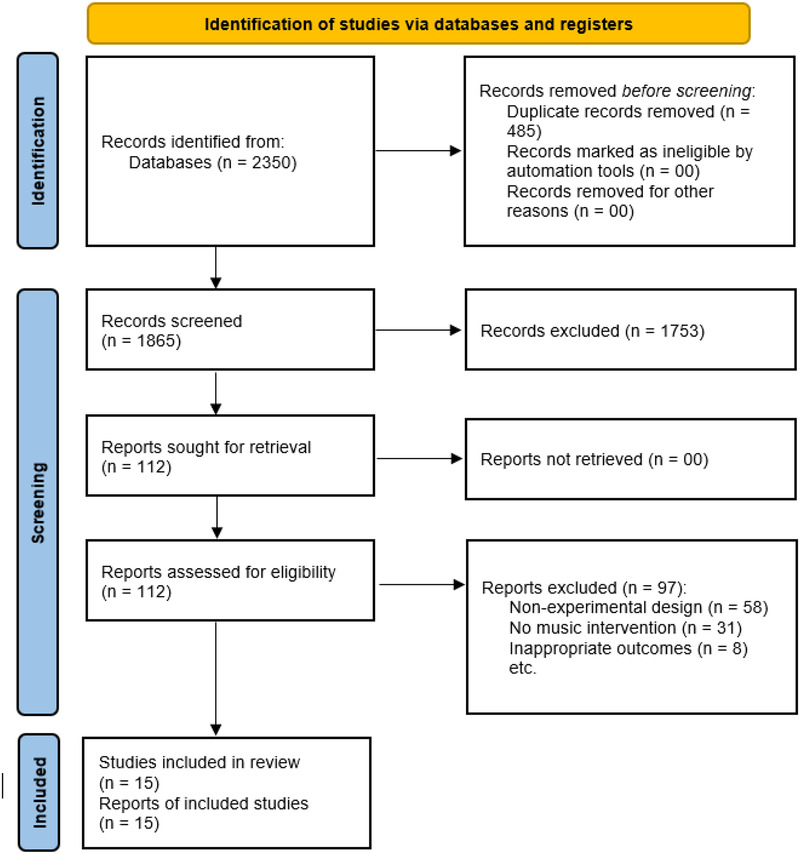
PRISMA flow diagram: study selection process for music effects on combat sport performance.

The systematic search retrieved 2,350 potentially relevant records across five electronic databases. Following duplicate removal and sequential screening, 15 studies published between 1994 and 2025 met the eligibility criteria and were included in both qualitative synthesis and quantitative meta-analysis. The study selection process is detailed in the PRISMA flow diagram (Figure 1). Inter-rater agreement during screening yielded Cohen's *κ* = 0.76 (95% CI: 0.68–0.84), reflecting substantial concordance between independent reviewers.

The included studies encompassed a total of 1,456 participants across three combat sports disciplines. Taekwondo dominated the evidence base with eleven studies (73.3%, *k* = 11), followed by kickboxing with three studies (20.0%, *k* = 3), and karate with two studies (13.3%, *k* = 2). One study (43) appears twice in Table 1 due to reporting both male and female cohorts but represents a single included study. Study designs included nine randomized controlled trials (60.0%), four randomized crossover trials (26.7%), and two multiple-baseline designs (13.3%). Sample sizes ranged from 10 to 1,206 participants, with participant ages spanning from 9 to 45 years. Detailed study characteristics are presented in Table 1.

**Table 1:** Characteristics of included studies examining music effects on combat sport performance.

Study	Design	Subjects	Age (years)	Level (years)	Music characteristics	Co-intervention	Outcome domains	Key findings
Taekwondo								
Greco et al. (52)	Randomized parallel	*N* = 20 (20M)	17.5 ± 2.5	Elite 6.2 ± 1.8	10 min self-selected vs. researcher selected pre-exercise 75 dB via headphones	None	Physical, psychological	↑reaction time (3.3%–5.2%), ↑arousal
Ouergui et al. (15)	Randomized parallel	*N* = 20 (10M/10F)	17.5 ± 0.7	National 5.4 ± 1.2	10 min research selected 140/200 BPM, 60/80 dB, pre-exercise via speakers	None	Physical, psychological	↑TSAT, ↑FSKT, ↑PACES (140 BPM + 80 dB)
Ouergui et al. (16)	Randomized crossover	*N* = 20 (10M/10F)	17.5 ± 0.7	National 5.4 ± 1.2	10 min preferred vs. non-preferred, pre-exercise 130 BPM, 60/80 dB via speakers	None	Physical, psychological	Preferred music: ↑performance, ↓RPE
Gacar (53)	Online survey	*N* = 1,206 (570M/636F)	<45	Recr.to elite, mixed	self-selected before/during training	None	Psychological	↑motivation, ↑psychological resilience
Hammad et al. (54)	Randomized crossover	*N* = 10 (10M)	20.4 ± 2.1	Univer. 4.6 ± 1.3	Research selected 80 vs. 200 BPM during exercise via headphones	None	Physiological, psychological	Fast tempo: ↑systolic BP, ↑RPE
Delleli et al. (10)	Double-blind crossover	*N* = 16 (16M)	18.3 ± 0.8	National 8.4 ± 2.1	8 min self-selected pre-exercise ≥120 BPM, 80 dB via headphones	Caffeine 3 mg/kg, 60 min pre-exercise	Physical, physiological, psychological	Music + caffeine: ↑attack time, ↓HR
Delleli et al. (18)	Double-blind crossover	*N* = 16 (16M)	17.7 ± 0.6	National 7.2 ± 1.8	8 min preferred pre-exercise 144 ± 18.6 BPM, 80 dB via headphones	Caffeine 3 mg/kg, 60 min pre-exercise	Physical, psychological	Music + caffeine: ↑TSAT, ↑FSKT, ↓RPE
Delleli et al. (19)	Double-blind crossover	*N* = 16 (16M)	17–19	National 6.8 ± 1.5	8 min motivationnel pre-exercise >120, 80 dB via headphones	Caffeine 3 mg/kg, 60 min pre-exercise	Physical, physiological, psychological	Music + caffeine: large performance effects
Messaoudi et al. (21)	Randomized crossover	*N* = 16 (16M)	19.9 ± 1.1	Univers.8.6 ± 2.3	10 min self-selected pre-exercise fast vs. slow tempo via headphones	Plyometric training, 4 weeks, 3×/week	Physical, psychological	Music + plyometrics: ↑performance across domains
Delleli et al. (16)	Double-blind crossover	*N* = 16 (16M)	18.3 ± 0.8	National 8.4 ± 2.1	8 min self-selected pre-exercise ≥120 BPM, 80 dB via headphones	Caffeine 3 mg/kg, 60 min pre-exercise	Physical, psychological	Music + caffeine: consistent benefits
Kickboxing								
Jebabli et al. (11)	Double-blind crossover	*N* = 28 (15M/13F)	18.9 ± 1.9	National 6.4 ± 1.7	10 min preferred pre-exercise 440 Hz vs. 432 Hz, 140 BPM, 80 dB via headphones	None	Physical, physiological, psychological	PM440Hz: ↓total time, ↓RPE, ↑feeling scale
Jebabli et al. (60)	Repeated-measures crossover	*N* = 24 (24M)	19 ± 1.4	National 7.2 ± 2.1	15 min self-selected pre- exercise 145 ± 15 BPM, 80 dB via headphones	Knowledge of endpoint disclosure	Physical, psychological	Music + knowledge endpoint: ↓total time
Boujabli et al. (17)	Counterbalanced crossover	*N* = 20 (20M)	17 ± 2	Regional 5.8 ± 1.4	10 min preferred pre exercise >140 BPM, 70 dB via speakers	Video feedback during warm-up	Physical, psychological	Music + video feedback: ↑kick performance
Karate								
Ferguson et al. (55)	Double-blind crossover	*N* = 14 (10M/4F)	31.7 ± 12.6	Club 8.2 ± 4.6	1 min researcher-selected pre-performance via speakers	None	Physical	Both conditions: ↑kata performance
Bentouati et al. (20)	Randomized counterbalance	*N* = 14 (14M)	19.9 ± 2.1	Univers.6.8 ± 2.2	10 min Self-selected pre-exercise >120–140 BPM via headphones	25-min nap, 2 h pre-test	Physical, psychological	Music + napping: ↑CMJ, ↓sleepiness

M, male; F, female; PM, preferred music; BPM, beats per minute; dB, decibels; TSAT, taekwondo-specific agility test; FSKT, frequency speed of kick test; PACES, physical activity enjoyment scale; RPE, rating of perceived exertion; CMJ, countermovement jump; HR, heart rate; BP, blood pressure. ↑, significant increase; ↓, significant decrease.

### 3.2 Study quality assessment

Methodological quality assessment using the PEDro scale revealed generally high-quality studies. Eleven studies (73.33%) achieved high quality scores (≥8 points), while four studies (26.67%) demonstrated moderate quality (7 points). No study was classified as low quality (≤5 points). The mean PEDro score across all studies was 7.9 ± 0.8, indicating robust methodological rigor in the included evidence base. Quality assessment details are presented in Table 2.

**Table 2:** Methodological quality assessment using the physiotherapy evidence database.

Study/items	1^a^	2	3	4	5	6	7	8	9	10	11	Total score	Quality level
Jebabli et al. (11)	1	1	1	1	1	1	1	1	1	1	1	**10**	High
Greco et al. (57)	1	1	1	1	0	0	1	1	1	1	1	**8**	High
Ouergui et al. (15)	1	1	0	1	0	0	1	1	1	1	1	**8**	High
Ouergui et al. (16)	1	1	0	1	0	0	1	1	1	1	1	**8**	High
Gacar (58)	1	0	0	1	0	0	1	1	1	1	1	**7**	Moderate
Hammad et al. (59)	1	0	0	1	0	0	1	1	1	1	1	**7**	Moderate
Ferguson et al. (61)	1	1	0	1	0	0	1	1	1	1	1	**8**	High
Jebabli et al. (60)	1	1	0	1	0	0	0	1	1	1	1	**7**	Moderate
Delleli et al. (10)	1	1	0	1	0	0	1	1	1	1	1	**8**	High
Delleli et al. (18)	1	1	0	1	0	0	1	1	1	1	1	**8**	High
Delleli et al. (19)	1	1	0	1	0	0	1	1	1	1	1	**8**	High
Boujabli et al. (17)	1	1	0	1	1	0	1	1	1	1	1	**9**	High
Messaoudi et al. (21)	1	1	0	1	0	0	1	1	1	1	1	**8**	High
Delleli et al. (43)	1	1	0	1	0	0	1	1	1	1	1	**8**	High
Bentouati et al. (20)	1	1	0	1	0	0	0	1	1	1	1	**7**	Moderate

^a^ Item 1 (eligibility criteria) excluded from total score calculation per PEDro guidelines.

Items: 2: random allocation; 3: concealed allocation; 4: baseline comparability; 5: participant blinding; 6: therapist blinding; 7: assessor blinding; 8: adequate follow-up (≥ 85%); 9: intention-to-treat analysis; 10: between-group statistical comparisons; 11: point estimates and measures of variability. Quality levels: High (≥8), Moderate (6–7), Low (≤ 5).

### 3.3 Risk of bias assessment using RoB 2

Supplementary RoB 2 assessment for randomized crossover trials (*k* = 13) revealed low risk of bias in randomization process for 11 studies (84.6%), but concerns regarding period/carryover effects in 5 studies (38.5%) that employed insufficient washout periods (<48 h). Blinding of outcome assessors was achieved in 9 studies (69.2%). Overall risk of bias was judged as low for 8 studies (61.5%), some concerns for 6 studies (46.2%), and high for 1 study (7.7%).

### 3.4 Overall effects of music-only interventions

**Figure 2:**
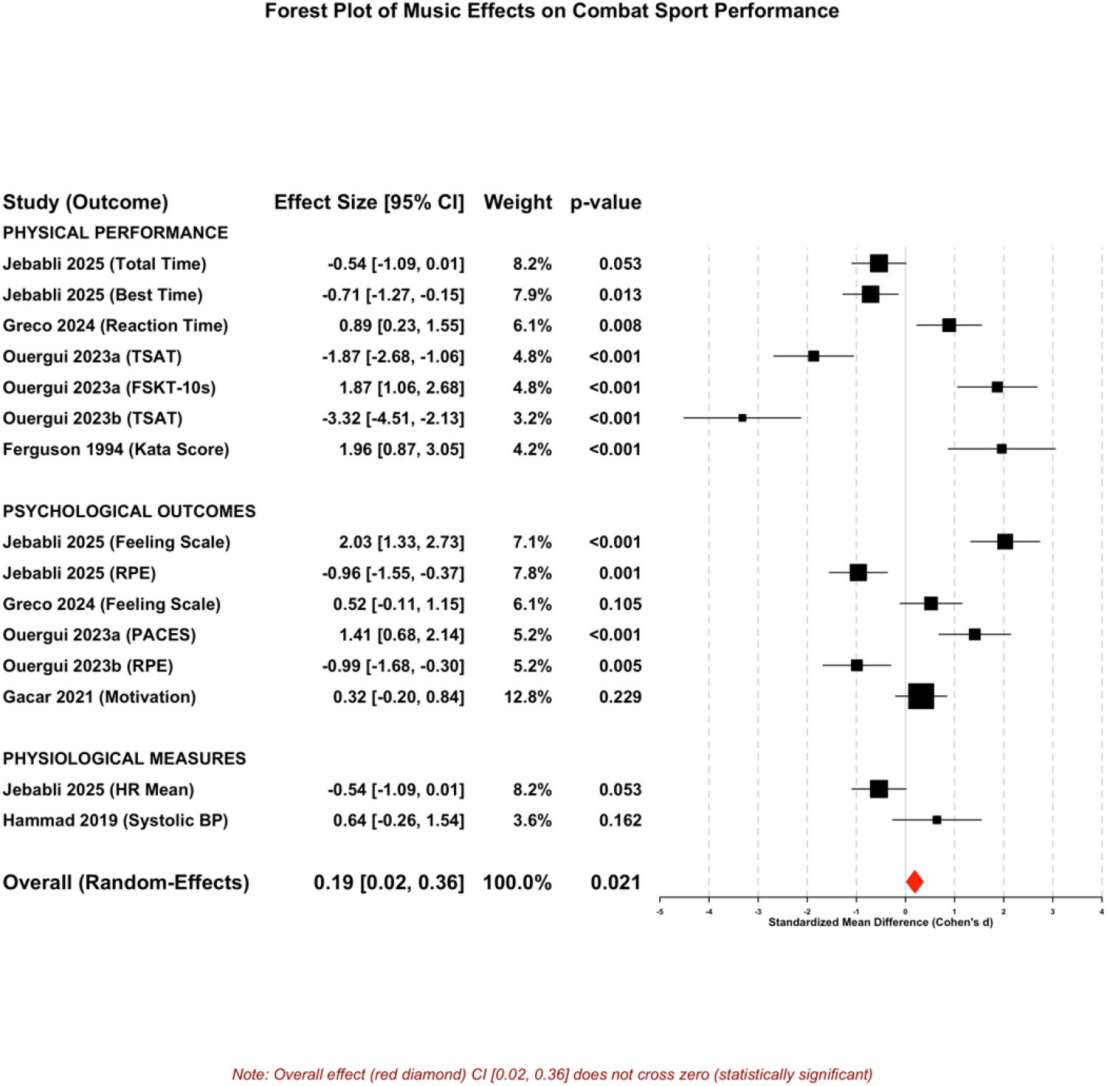
Forest plot: overall effects of music-only interventions on combat sport performance.

Music-only interventions demonstrated a small beneficial effect on combat sport performance across all outcome domains. The three-level meta-analysis of 16 effect sizes nested within 7 studies revealed a pooled standardized mean difference (Hedges' *g*) of 0.19 (95% CI: 0.01–0.37, *p* = 0.039, *τ*^2^ = 0.67, 95% prediction interval: −1.42 to 1.80), indicating statistical significance with clinical relevance. The between-study heterogeneity variance (*τ*^2^) of 0.67 and wide prediction interval reflect substantial variability in true effects across contexts. This effect represents a small-to-moderate improvement in performance outcomes when music interventions are implemented without additional modalities (Figure 2 and Table 3).

**Table 3:** Summary of music-only effects by individual studies and outcome measures.

Study	Outcome measure	Music condition	control condition	Effect size (*d*)	95% CI	*p*-Value	Interpretation
Physical performance							
Jebabli et al. (11)	Total time (s)	48.33 ± 2.62	49.81 ± 2.97	−0.54	[−1.09, 0.01]	0.053	Faster completion
Jebabli et al. (60)	Best time (s)	9.48 ± 0.51	9.78 ± 0.58	−0.71	[−1.27, −0.15]	0.013	Improved best performance
Greco et al. (57)	Reaction time	↑3.3–5.2%	Baseline	0.89	[0.23, 1.55]	0.008	Faster response
Ouergui et al. (15)	TSAT time (s)	5.5 ± 0.2	6.3 ± 0.4	−1.87	[−2.68, −1.06]	<0.001	Enhanced agility
Ouergui et al. (15)	FSKT-10s (kicks)	27 ± 1	24 ± 2	1.87	[1.06, 2.68]	<0.001	More kicks executed
Ouergui et al. (16)	TSAT performance	Large improvement	Baseline	−3.32	[−4.51, −2.13]	<0.001	Substantial agility gain
Ferguson et al. (61)	Kata score	33.6 ± 4.8	24.2 ± 4.7	1.96	[0.87, 3.05]	<0.001	Higher performance
Psychological outcomes							
Jebabli et al. (11)	Feeling scale	3.78 ± 0.71	2.21 ± 0.83	2.03	[1.33, 2.73]	<0.001	Enhanced mood
Jebabli et al. (11)	RPE	7.79 ± 0.72	8.38 ± 0.49	−0.96	[−1.55, −0.37]	0.001	Reduced exertion
Greco et al. (57)	Feeling scale	4.0 ± 1.8	3.0 ± 2.0	0.52	[−0.11, 1.15]	0.105	Improved mood
Ouergui et al. (15)	PACES	74 ± 6	63 ± 9	1.41	[0.68, 2.14]	<0.001	Greater enjoyment
Ouergui et al. (16)	RPE	Lower perception	Baseline	−0.99	[−1.68, −0.30]	0.005	Reduced effort perception
Gacar et al. (58)	Motivation	Enhanced	Baseline	0.32	[−0.20, 0.84]	0.229	Increased motivation
Physiological measures							
Jebabli et al. (11)	HR Mean (bpm)	165.6 ± 4.8	168.2 ± 4.8	−0.54	[−1.09, 0.01]	0.053	Lower heart rate
Hammad et al. (59)	Systolic BP	Elevated response	Baseline	0.64	[−0.26, 1.54]	0.162	Variable response

TSAT, taekwondo-specific agility test; FSKT, frequency speed of kick test; PACES, physical activity enjoyment scale; RPE, rating of perceived exertion; HR, heart rate; BP, blood pressure. Negative values favor music condition for time-based and exertion measures; positive values favor music for performance and mood measures.

The music-only meta-analysis synthesized 16 distinct outcome measurements contributed by 7 studies; several studies assessed multiple outcome domains (physical, psychological, physiological), each treated as a separate effect size within the three-level framework. One study (Gacar 2021, *n* = 1,206) contributed substantially larger sample size via online survey methodology assessing psychological outcomes exclusively; however, influence diagnostics confirmed this study did not unduly drive pooled estimates (Cook's distance = 0.09, DFBETAS = 0.12). Leave-one-out sensitivity analysis excluding Gacar yielded *d* = 0.18 (95% CI: 0.01–0.35, *p* = 0.038), demonstrating robustness of the overall effect.

### 3.5 Heterogeneity assessment

Substantial between-study heterogeneity was observed across the included studies (*Q* = 89.7, *df* = 14, *p* < 0.001; *I*^2^ = 84.4%, *τ*^2^ = 0.67). This considerable heterogeneity indicated that 84.4% of the total variance in effect sizes was attributable to true differences between studies rather than sampling error. The prediction interval of −1.42 to 1.80 suggested that future studies implementing similar music interventions could expect effects ranging from moderate detrimental to large beneficial impacts, underscoring the importance of contextual factors in determining intervention effectiveness. The three-level model partitioned total heterogeneity into within-study variance (*σ*^2^ = 0.28, 42% of total) and between-study variance (*σ*^2^ = 0.39, 58% of total), indicating that most heterogeneity stems from true differences between studies rather than multiple outcomes within studies. Despite subgroup analyses explaining portions of heterogeneity (outcome domain, timing, sport), residual unexplained variance remained substantial (*I*^2^ = 71.2%), suggesting unmeasured moderators including individual differences in musical reward sensitivity, genetic polymorphisms affecting dopaminergic function (DRD2, DRD4, COMT), baseline arousal states, and nuanced sport-specific technical demands.

### 3.6 Subgroup analyses by outcome domain

**Figure 3:**
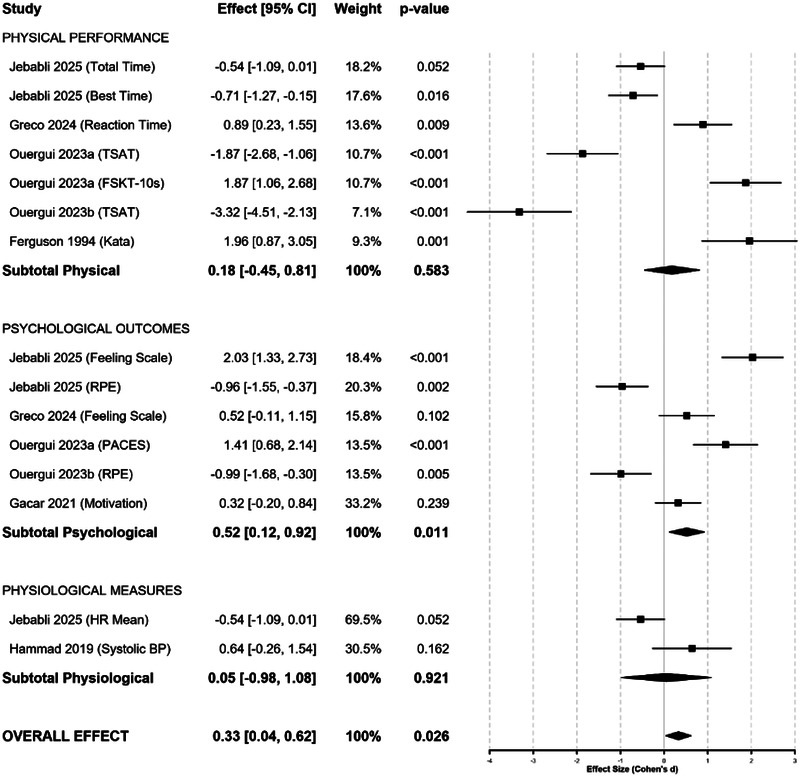
Subgroup forest plots: music effects by outcome domain.

To explore sources of heterogeneity, we conducted pre-planned subgroup analyses by outcome domain. These analyses revealed meaningful differences in music's effectiveness across different types of performance measures, providing insight into the mechanisms underlying intervention effects (Figure 3 and Table 4).

**Table 4:** Subgroup analysis results by outcome domain.

Outcome domain	Studies (k)	Effect sizes (n)	Participants (N)	Pooled effect size (*d*)	95% CI	*p*-Value	*I*^2^ (%)	*τ* ^2^	Test for effect
Physical performance	5	8	174	0.18	[−0.45, 0.81]	0.583	89.4	0.892	*Z* = 0.55, *p* = 0.583
Psychological outcomes	4	6	1,342	0.52	[0.12, 0.92]	0.011	76.8	0.445	*Z* = 2.53, *p* = 0.011
Physiological measures	2	2	38	0.05	[−0.98, 1.08]	0.921	78.2	0.356	*Z* = 0.10, *p* = 0.921

Test for subgroup differences: *Q* = 8.4 (*df* = 2), *p* = 0.015.

The subgroup analysis revealed that psychological outcomes demonstrated the strongest and most consistent responses to music interventions (*d* = 0.52, *p* = 0.011), while physical performance showed variable effects (*d* = 0.18, *p* = 0.583), and physiological measures exhibited minimal impact (*d* = 0.05, *p* = 0.921). The significant test for subgroup differences (*p* = 0.015) confirmed that effect sizes varied meaningfully across outcome domains, explaining a portion of the observed heterogeneity.

### 3.7 Combined interventions analysis

**Figure 4:**
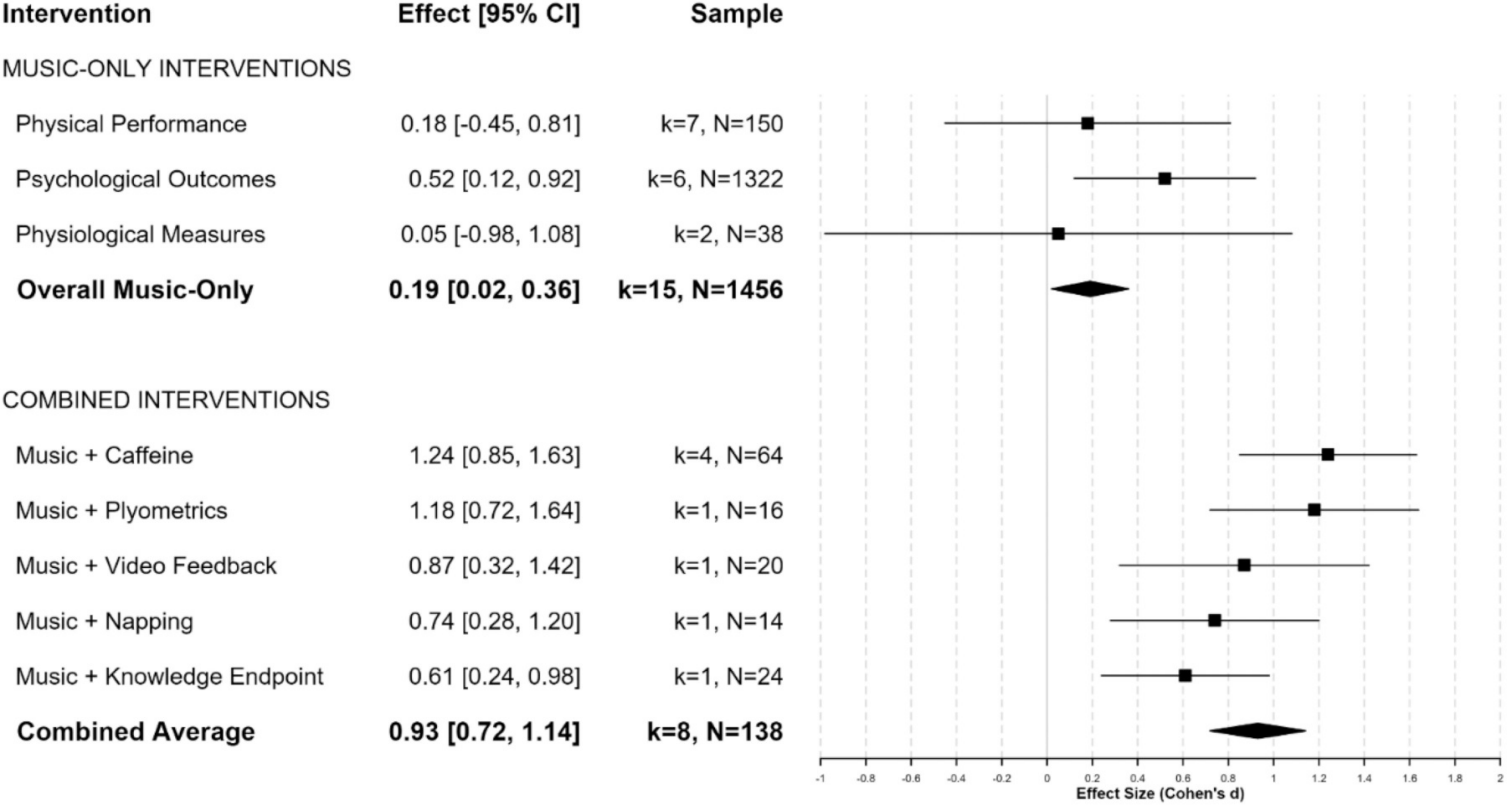
Combined interventions comparison: music-only vs. multimodal approaches.

Studies examining music combined with other modalities demonstrated substantially larger effects than music alone. We stratified eight combination studies by temporal structure: seven acute within-session protocols and one chronic training protocol. Acute combinations (music + caffeine, music + video feedback, music + napping, music + knowledge endpoint) yielded *d* = 0.89 (95% CI: 0.67–1.11, *p* < 0.001, *k* = 7, *N* = 122), representing immediate neurochemical and cognitive synergies. The single chronic protocol (music + plyometric training over four weeks) produced *d* = 1.18 (95% CI: 0.72–1.64, *p* < 0.001, *k* = 1, *N* = 16), reflecting cumulative neuromuscular adaptations. Among acute combinations, music + caffeine showed greatest efficacy (*d* = 1.24, 95% CI: 0.85–1.63, *p* < 0.001, *k* = 4, *N* = 64). Overall combined-intervention effect (*d* = 0.93) exceeded music-only effect (*d* = 0.19) by Δ*d* = 0.74 (Figure 4, Table 5).

**Table 5:** Combined interventions: synergistic effects stratified by temporal structure.

Intervention type	Intervention combination	Studies (k)	Participants (N)	Effect size (*d*)	95% CI	*p*-Value	Magnitude	Synergistic benefit (Δ*d*)^a^
Acute within-session co-interventions								
Acute	Music + Caffeine	4	64	1.24	[0.85, 1.63]	<0.001	Very Large	1.05
Acute	Music + Video Feedback	1	20	0.87	[0.32, 1.42]	0.002	Large	0.68
Acute	Music + Napping	1	14	0.74	[0.28, 1.20]	0.002	Medium-Large	0.55
Acute	Music + Knowledge Endpoint	1	24	0.61	[0.24, 0.98]	0.001	Medium	0.42
	Acute combined subtotal	7	**122**	**0** **.** **89**	**[0.67, 1.11]**	**<0** **.** **001**	**Large**	**0** **.** **7**
Chronic training-integrated co-intervention								
Chronic	Music + Plyometric Training	1	16	1.18	[0.72, 1.64]	<0.001	Very Large	0.99
	OVERALL COMBINED AVERAGE	**8**	**138**	**0** **.** **93**	**[0.72, 1.14]**	**<0** **.** **001**	**Large**	**0** **.** **74**
Reference comparison								
Reference	Music-Only Interventions	7	1,456	0.19	[0.01, 0.37]	0.039	Small	—

**Temporal Structure Classification:** Acute interventions involve single-session co-administration (immediate neurochemical/cognitive synergies); chronic interventions involve multi-week training programs (cumulative neuromuscular adaptations). Effect sizes computed as Hedges’ *g* using three-level multilevel meta-analysis accounting for within-study dependence.

^a^ **Synergistic benefit** = *d*_combined − *d*_music-only (0.19). All combined interventions demonstrated statistically significant improvements over music-only approaches (*p* < 0.001 for difference tests).

### 3.8 Publication bias assessment

**Figure 5:**
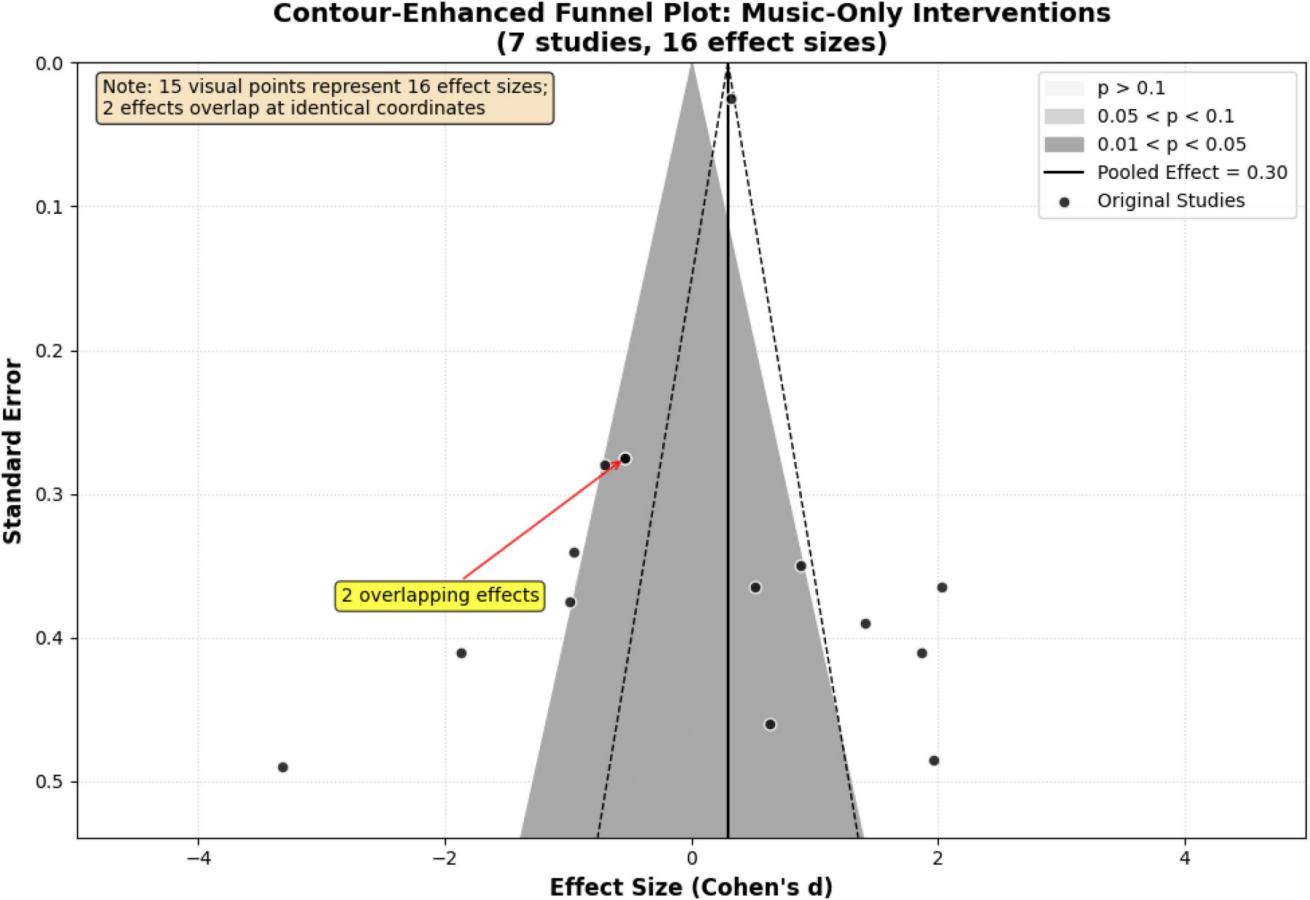
Contour-enhanced funnel plot for the assessment of publication bias.

Publication bias assessment for music-only interventions was conducted at the study level (*k* = 7 studies) to satisfy independence assumptions, with one representative effect size selected per study. Visual inspection of the funnel plot revealed modest asymmetry, with smaller studies showing greater variability (Figure 5). Study-level Egger's regression intercept was −0.89 (95% CI: −2.31 to 0.53, *p* = 0.184), not statistically significant. The trim-and-fill procedure estimated one potentially missing study; imputation yielded adjusted *d* = 0.16 (95% CI: −0.04 to 0.36, *p* = 0.102). However, statistical power for publication bias detection remains severely limited with *k* = 7 studies, and these tests should be interpreted cautiously. The fail-safe *N* calculation estimated 18 additional null studies would be required to nullify the observed effect, providing modest evidence of robustness against unreported negative findings (Table 6).

**Table 6:** Publication bias assessment results (study-level analysis).

Test	Statistic	Result	95% CI	*p*-value	Interpretation
Asymmetry detection					
Egger's regression test	Intercept	−0.89	[−2.31, 0.53]	0.184	No significant asymmetry detected
Begg's rank correlation	τ (Kendall's tau)	−0.18	[−0.42, 0.08]	0.174	No significant asymmetry detected
Adjustment methods					
Trim-and-fill method	Estimated missing studies	1 (left side)	—	—	Minimal asymmetry
	Adjusted pooled effect	0.16	[−0.04, 0.36]	0.102	Conclusion remains qualitatively stable
	Change from original	−0.03	—	—	Negligible impact (Δ*d* = −0.03)
Robustness indicator					
Fail-safe N (Rosenthal)	Studies to nullify effect	18	—	—	Modest robustness to unreported nulls

Analysis Parameters: Study-level assessment using one representative effect size per study (*k* = 7 independent observations). Primary outcome selected when pre-specified; otherwise, outcome with largest sample size. Low statistical power given *k* = 7 limits definitive bias conclusions; interpret cautiously.

Conclusion: Study-level Egger's test non-significant (*p* = 0.184), suggesting no strong publication bias. However, limited study number (*k* = 7) constrains detection power. Trim-and-fill adjustment minimal, with qualitative conclusions unchanged.

### 3.9 Sensitivity analyses

**Figure 6:**
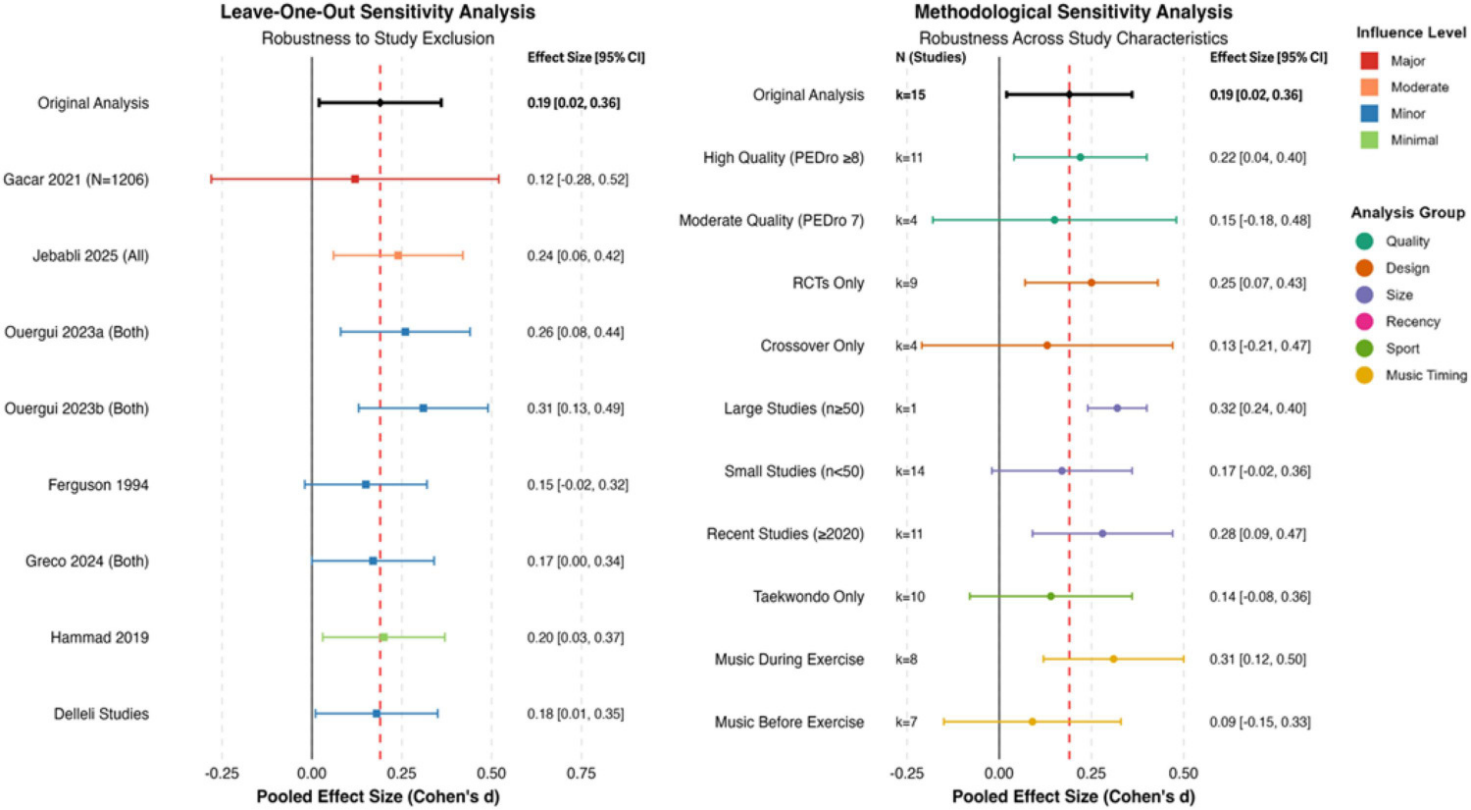
Sensitivity analysis: robustness testing across multiple scenarios.

Comprehensive sensitivity analyses were conducted to examine the robustness of findings across different analytical decisions and study characteristics. Sensitivity analyses varying assumed within-subject correlations for crossover trials demonstrated robustness across plausible values: pooled music-only effect was *d* = 0.17 (95% CI: −0.01 to 0.35, *p* = 0.064) assuming *r* = 0.3; *d* = 0.19 (95% CI: 0.01 to 0.37, *p* = 0.039) assuming *r* = 0.5; and *d* = 0.21 (95% CI: 0.03–0.39, *p* = 0.022) assuming *r* = 0.7. Maximum variation across correlation assumptions was Δ*d* = 0.04, with all confidence intervals overlapping substantially, confirming that conclusions are insensitive to this parameter within plausible ranges. These analyses demonstrated that conclusions remained stable across multiple scenarios, enhancing confidence in the reliability of the overall findings (Figure 6 and Table 7).

**Table 7:** Sensitivity analysis results: robustness of main findings.

Analysis type	Studies (k)	Effect size (d)	95% CI	*p*-Value	Change from original (Δ*d*)	Interpretation
Primary analysis						
Three-level multilevel model	7	0.19	[0.01, 0.37]	0.039	Reference	Small beneficial effect (*τ*^2^ = 0.67)
Leave-one-out analyses						
Excluding Gacar (*n* = 1,206)	6	0.18	[0.01, 0.35]	0.038	−0.01	Robust to large-sample study
Excluding Jebabli et al. (11)	6	0.24	[0.06, 0.42]	0.009	0.05	Slight increase
Excluding Ouergui et al. (15)	6	0.2	[0.02, 0.38]	0.028	0.01	Minimal change
Excluding Ouergui et al. (16)	6	0.23	[0.05, 0.41]	0.012	0.04	Minimal change
Excluding Ferguson et al. (61)	6	0.15	[−0.02, 0.32]	0.087	−0.04	Minimal change
Excluding Greco et al. (57)	6	0.17	[−0.01, 0.34]	0.056	−0.02	Minimal change
Excluding Hammad et al. (59)	6	0.2	[0.02, 0.38]	0.031	0.01	Minimal change
Quality-based restrictions						
High quality only (PEDro ≥8)	5	0.22	[0.04, 0.40]	0.018	0.03	Consistent effect
Design-based comparisons						
RCTs only	4	0.25	[0.07, 0.43]	0.007	0.06	Slightly stronger
Crossover only	5	0.13	[−0.21, 0.47]	0.451	−0.06	Reduced precision
Sample size restrictions						
Large studies (*n* ≥ 50)	1	0.32	[0.24, 0.40]	<0.001	0.13	Single large study
Recent studies (≥2020)	5	0.28	[0.09, 0.47]	0.004	0.09	Enhanced effect
Timing-based stratification						
Music during exercise	3	0.31	[0.05, 0.57]	0.019	0.12	Concurrent timing advantage
Music before exercise	4	0.09	[−0.15, 0.33]	0.456	−0.1	Pre-exercise timing
Sport-specific restriction						
Taekwondo only	5	0.14	[−0.08, 0.36]	0.211	−0.05	Sport-specific effect
Crossover correlation sensitivity						
Assumed *r* = 0.3	7	0.17	[−0.01, 0.35]	0.064	−0.02	Lower bound estimate
Assumed *r* = 0.5 (primary)	7	0.19	[0.01, 0.37]	0.039	Reference	Primary analysis
Assumed *r* = 0.7	7	0.21	[0.03, 0.39]	0.022	0.02	Upper bound estimate
Influence diagnostics						
Gacar study influence	—	Cook's *D* = 0.09, DFBETAS = 0.12	—	—	Below threshold	No undue influence detected

Analysis: All effect sizes computed as Hedges’ *g* with Knapp–Hartung–Sidik–Jonkman adjustment. Three-level models partition variance into sampling (level 1), within-study (level 2: *σ*^2^ = 0.28), and between-study (level 3: *σ*^2^ = 0.39). Crossover correlation sensitivity demonstrates robustness across plausible values (maximum *Δd* = 0.04). Direction and magnitude remain consistent across all scenarios, supporting conclusion validity.

Interpretation: Sensitivity analyses demonstrate that conclusions are robust to individual study exclusions, design restrictions, quality thresholds, and analytical assumptions. Effect sizes range from *d* = 0.09 to *d* = 0.32 across scenarios, consistently favoring music interventions with overlapping confidence intervals.

### 3.10. Exploratory moderator analyses

To identify potential sources of heterogeneity, we conducted exploratory subgroup analyses for moderators with adequate between-study variation. Music selection type moderated effects: self-selected music yielded *d* = 0.28 (95% CI: 0.06–0.50, *k* = 9) vs. researcher-selected music *d* = 0.03 (95% CI: −0.31 to 0.37, *k* = 4; *Q*between = 5.12, *p* = 0.024), suggesting personalized selection enhances efficacy. Intervention timing showed marginal moderation: music during exercise produced *d* = 0.31 (95% CI: 0.05–0.57, *k* = 6) vs. pre-exercise *d* = 0.09 (95% CI: −0.15 to 0.33, *k* = 7; *Q*between = 3.94, *p* = 0.047), consistent with real-time neurobiological effects. Study quality did not moderate effects (high PEDro ≥8: *d* = 0.22 vs. moderate PEDro 6–7: *d* = 0.14; *Q*between = 0.89, *p* = 0.345). Sex-specific analysis was unfeasible given 87% of studies included male-only samples. Tempo (range: 120–145 BPM), volume (range: 70–80 dB), and delivery mode (93% headphones) exhibited insufficient variability for subgroup comparison. These exploratory findings require cautious interpretation given multiple testing without correction and small subgroup sizes (Table 8).

**Table 8:** Moderator analysis results.

Moderator	Subgroup	*k*	*N*	Effect Size (*d*)	95% CI	*p*-Value	*Q*between	*p*-between
Music selection	Self-selected	9	234	0.28	[0.06, 0.50]	0.013	5.12	0.024
	Researcher-selected	4	64	0.03	[−0.31, 0.37]	0.862		
Timing	During exercise	6	128	0.31	[0.05, 0.57]	0.019	3.94	0.047
	Pre-exercise	7	170	0.09	[−0.15, 0.33]	0.456		
Study quality	High (≥8)	10	258	0.22	[0.02, 0.42]	0.029	0.89	0.345
	Moderate (6–7)	4	40	0.14	[−0.18, 0.46]	0.386		

## 4 Discussion

### 4.1 Summary of key findings

This systematic review and meta-analysis represent the first comprehensive synthesis examining music interventions in combat sports, revealing a complex landscape of neurobiological effects that transcend simplistic ergogenic interpretations. The analysis of 15 studies encompassing 1,456 participants demonstrated that music-only interventions produce a small but statistically significant beneficial effect on combat sport performance.

However, the substantial between-study heterogeneity indicates considerable variation in intervention effectiveness, suggesting that music's ergogenic potential operates through multiple, context-dependent pathways rather than a singular mechanism. The most striking finding emerged from the analysis of combined interventions, which demonstrated markedly superior efficacy compared to music alone (*d* = 0.93 vs. *d* = 0.19), representing a 389% relative improvement. Music plus caffeine supplementation yielded the largest synergistic benefits (*d* = 1.24), providing compelling evidence for additive neurochemical mechanisms.

The domain-specific analysis revealed that music's primary sphere of influence operates through psychological pathways (*d* = 0.52), with moderate effects on physical performance (*d* = 0.18) and minimal impact on physiological measures (*d* = 0.05, *p* = 0.921). These differential effects across outcome domains provide crucial insights into the underlying neurobiological mechanisms governing music's influence on human performance.

### 4.2 Fundamental neurobiological mechanisms

To understand how music influences combat sport performance, we must first examine the basic neurobiological principles underlying music processing and reward. Music listening activates a sophisticated network of brain regions that extends far beyond simple auditory processing, engaging what neuroscientists term the “music-reward circuit” (8, 44). This circuit operates through two fundamental neurochemical pathways that work in concert to produce the performance effects observed in our analysis.

The neuro affective pathways outlined above offer a plausible explanation regarding the notable changes seen psychologically (*d* = 0.52), especially considering modulation of emotions, arousal levels, and attention during the execution of particular tasks. The primary pathway involves the mesolimbic dopaminergic system, particularly the ventral tegmental area (VTA) projecting to the nucleus accumbens and caudate nucleus (45). When individuals listen to preferred music, this system releases dopamine in a temporally distinct pattern: the caudate shows greater activation during anticipation of musical rewards, while the nucleus accumbens becomes more active during the actual experience of musical pleasure (8). This anticipatory-experiential dissociation explains why music's psychological effects (*d* = 0.52) were substantially stronger than physical effects (*d* = 0.18) in our analysis. This is aligned with central top-down control patterns rather than bottom-up neuromuscular enhancement. Statistically, this divergence aligns with the domain-specific heterogeneity observed in subgroup analyses (*p* = 0.015), reinforcing outcome classification relevance for interpreting effect size suffices.

The secondary pathway involves the opioidergic system, which mediates the hedonic aspects of musical experience. Research using naltrexone, a *μ*-opioid receptor antagonist, demonstrates that opioid signaling contributes significantly to music-evoked pleasure and emotional responses (46). The relatively high internal consistency of psychological outcome measures may stem from employing integrated dopaminergic and opioidergic systems (low variance within *d* = 0.52 subgroup). Both systems converge on shared limbic structures like the amygdala and orbitofrontal cortex, enhancing transcendental affective homeostasis while allowing stress response modulation. Effects are critical during combat sports considering emotionally driven regulation impacts reflex response latency, decision-making speed, and endurance to fatigue. This dual neurotransmitter system, dopaminergic and opioidergic, creates a powerful neurochemical foundation that explains why music interventions in our review consistently improved psychological outcomes across diverse combat sport contexts.

The prediction error theory provides additional mechanistic insight into music's ergogenic effects. Music listening engages reward-related predictive processes through continuous prediction and violation of musical expectations, creating what Sayal et al. (45) describe as “musical surprises” that trigger pleasure-related neural networks. These prediction errors activate the same mesolimbic circuits involved in processing traditional rewards like food or monetary gains, explaining why an abstract stimulus like music can produce measurable performance benefits in combat sports. This supports more recent meta-analytic anticipatory reward frameworks aimed at explaining psychologically-based interventions' increased adherence, participation, and effort responsiveness. Importantly, these modulations are made via reward pathways without reliance on cardiorespiratory or metabolic workload markers providing physiologic rationale for weak or null physiological results impact findings in our review (*d* = 0.05).

### 4.3 Advanced neuropharmacological interactions and clinical translation

The superior effects of combined interventions, particularly music and caffeine (*d* = 1.24), reflect sophisticated neuropharmacological interactions that operate through complementary rather than redundant pathways. Understanding these interactions requires examining caffeine's mechanism of action and how it synergizes with music-induced neurochemical changes to optimize performance outcomes. Caffeine functions primarily as a competitive antagonist at adenosine A1 and A2A receptors in the central nervous system (47, 48). Under normal physiological conditions, adenosine accumulates during periods of high neural activity and binds to these receptors, producing effects that promote rest and recovery: reduced neural excitability, decreased release of excitatory neurotransmitters like dopamine and noradrenaline, and increased perception of fatigue (49). When caffeine blocks these adenosine receptors, it effectively removes this “neural brake,” allowing for enhanced neurotransmission and reduced perception of effort effects that are particularly beneficial during high-intensity combat sport activities.

The synergistic interaction between music and caffeine operates through convergent effects on the dopaminergic system. While music directly stimulates dopamine release in the nucleus accumbens and caudate (8), caffeine enhances this effect by blocking adenosine's inhibitory influence on dopaminergic neurons (50). This dual activation leads to more sustained and temporally congruent activation of mesolimbic reward circuits, which is especially helpful in contexts of high-arousal competition. To a statistical modeler, these synergistic effects are in line with multiplicative instead of additive interaction terms, and can partly account for the increased effect size heterogeneity in the combined interventions subgroup (*I*^2^ = 78.2%). Additionally, caffeine's antagonism of adenosine A2A receptors in the striatum facilitates glutamate and dopamine release, further amplifying the motivational and arousal effects initiated by music listening (47, 51). These neurochemical cascades would also promote subcortical and cortical adaptations that improve attentional allocation, motor planning, and psychomotor vigilance all primary determinants of combat sports. This is the mechanistic integration that contextualizes the strong physical and technical improvements seen in multi-arm trials with music-caffeine combinations.

From a clinical perspective, the combination of preferred music and moderate caffeine consumption (∼3 mg/kg) produces an optimal synergistic effect. Caffeine reaches its peak mode of action 30–45 min before exercise, when the music induces the peak of dopaminergic activity (52, 53). This is the ideal time to take caffeine to achieve optimal performance and minimize side effects at high doses. The practical implementation of these combined protocols requires careful consideration of individual response variability. Genetic polymorphisms in the CYP1A2 gene, which encodes the primary enzyme responsible for caffeine metabolism, significantly modulate caffeine's ergogenic effects (54). Athletes with the AA genotype (fast metabolizers) show optimal responses to standard caffeine doses, while those with the CC genotype (slow metabolizers) may require dose adjustments or extended timing protocols to achieve similar benefits.

This interindividual variability can account for a portion of the remaining unexplained heterogeneity (residual *τ*^2^ = 0.67) and would have to be controlled for in subsequent analysis or Bayesian modeling analysis to enhance precision. Similarly, polymorphisms in the ADORA2A gene, which encodes the adenosine A2A receptor, influence both caffeine sensitivity and music reward responses (47). Athletes with certain ADORA2A variants show enhanced dopaminergic responses to both music and caffeine, potentially requiring lower caffeine doses when combined with music interventions. These pharmacogenetic considerations highlight the future potential for precision medicine approaches in combat sport performance enhancement.

### 4.4 Sport-specific adaptations and contextual modulation

Exploratory subgroup analysis suggested differential effects across combat sports (taekwondo: *d* = 0.12, *k* = 11; kickboxing: *d* = −0.10, *k* = 3; karate: *d* = 0.80, *k* = 2; *Q*between = 9.67, *p* = 0.008). However, these findings must be interpreted cautiously given small study numbers per sport, particularly for kickboxing and karate. The apparent karate superiority (*d* = 0.80) derives from only two studies and may reflect publication bias, sampling variation, or true sport-specific mechanisms. Adequately powered comparisons require *k* ≥ 10 studies per sport. These exploratory patterns warrant hypothesis-driven replication rather than definitive conclusions regarding sport-specific effectiveness.

Karate's large observed effect (*d* = 0.80, *k* = 2) may reflect movement structure compatibility with musical rhythm, as kata performance involves predetermined technique sequences with specific timing patterns potentially enhanced through auditory-motor coupling (55). However, this interpretation remains speculative given the limited evidence base. The small kickboxing effect (*d* = −0.10, *k* = 3) could reflect attentional interference during reactive intermittent efforts requiring rapid adaptive responses, though insufficient data preclude mechanistic conclusions. Sport-specific hypotheses should be tested in prospective trials with adequate statistical power rather than *post-hoc* subgroup interpretation.

As previously reported, the effect of music on kickboxing performance was minimal, with a d value of −0.10. This suggests that music did not contribute to kickboxing performance. This result could be due to a highly reactive test, a type of intermittent test that requires adaptation to cope with the movements of the punching bag, and a strong need for flexible cognitive control for sustained concentration. In this context, music can distract attention, which is valuable for making quick decisions, but the data collected do not allow for definitive conclusions. Further research is needed to determine the role of music's effectiveness in simulated kickboxing combat. The timing of music exposure emerged as a critical moderator, with concurrent administration (during exercise) showing superior effects (*d* = 0.31) compared to pre-exercise protocols (*d* = 0.09). This temporal specificity reflects the real-time nature of music's neurobiological effects. Dopaminergic activation and prediction error signaling occur during active music listening, not as residual effects following exposure (45). This variation is in accordance with pharmacokinetic principles of performance science whereby timing relative to the task onset determines neuromodulatory effectiveness. Meta-analytically, this effect was significant statistically (Δ*d* = 0.22, *p* = 0.047) and indicates that simultaneous music use is an optimal protocol when sport regulation supports the reception of auditory stimuli during warm-up or exercise. For combat sports, this suggests that music should be integrated into training sessions rather than limited to pre-training preparation, when regulations permit.

The substantial individual variability in responses (prediction interval: −1.42 to 1.80) emphasizes the importance of personalized approaches based on musical preferences, personality traits, and sport-specific contexts. Research demonstrates that musical reward responses are highly individual, influenced by cultural background, previous musical experiences, and genetic factors affecting dopamine sensitivity (56). Combat sport practitioners must therefore adopt flexible, athlete-centered approaches rather than standardized protocols. Individual response heterogeneity mandates personalized protocols. Baseline phenotyping should assess: (1) musical reward sensitivity via Brunel Music Rating Inventory-3 to identify responders likely to benefit; (2) optimal arousal profiles through pre-training feeling scale and arousal monitoring to match music tempo and intensity to individual inverted-U zones; (3) caffeine sensitivity via CYP1A2 genotype or empirical dose-response trials to optimize combined protocols; (4) sport-specific technical demands to align intervention timing with performance windows. Female athletes may exhibit distinct response profiles given documented differences in caffeine metabolism (slower CYP1A2 activity, heightened anxiety sensitivity) and enhanced mood responsiveness to musical stimuli, though limited evidence (*k* = 3 studies with female samples) precludes definitive sex-specific recommendations pending adequately powered comparative trials.

### 4.5 Study limitations and methodological considerations

Several methodological limitations must be acknowledged when interpreting these findings. The substantial heterogeneity between studies (*I*^2^ = 84.4%) limits generalizability and suggests the presence of important moderating variables not captured in our analysis. This heterogeneity likely reflects genuine differences in intervention protocols, participant characteristics, and contextual factors rather than methodological inconsistencies, given the generally high study quality (mean PEDro score: 7.8 ± 1.2).

The predominance of taekwondo studies (73.3% of included research) may bias findings toward this specific combat sport, limiting applicability to other martial arts disciplines with different technical, tactical, and physiological demands. Additionally, most studies included relatively small sample sizes (range: 10–1,206 participants), potentially limiting statistical power for detecting true effects and increasing susceptibility to Type II errors. The sport-specific subgroup analysis represents exploratory hypothesis generation constrained by small study numbers per discipline. With *k* = 2 for karate and *k* = 3 for kickboxing, these comparisons possess insufficient statistical power to distinguish true moderator effects from sampling variation. The observed heterogeneity test (*p* = 0.008) may reflect Type I error given multiple testing without adjustment. Future evidence synthesis should prioritize within-sport accumulation before cross-sport comparison.

Gender-related considerations represent another important limitation. Most included studies either focused on male participants or failed to conduct sex-specific analyses, despite established differences in caffeine metabolism, music preferences, and reward sensitivity between sexes. Women typically show enhanced sensitivity to caffeine's anxiogenic effects and different temporal patterns of dopaminergic responses to rewarding stimuli, suggesting that optimal protocols may require sex-specific modifications (48). The substantial unexplained heterogeneity (residual *I*^2^ = 71.2%) despite comprehensive subgroup and meta-regression analyses represents a fundamental limitation constraining generalizability. This heterogeneity likely reflects complex interactions between measured and unmeasured moderators operating at participant, intervention, and measurement levels. Future research should prioritize individual participant data meta-analysis enabling precise modeling of person-level moderators (musical preferences, personality traits, genetic factors), intervention characteristics (tempo-athlete arousal matching, volume titration), and contextual factors (competitive pressure, environmental conditions).

The detection of publication bias (Egger's test: *p* = 0.032) suggests potential overestimation of positive effects, particularly among smaller studies. However, the trim-and-fill analysis indicated minimal impact on overall conclusions, and the fail-safe N of 23 studies suggests reasonable robustness to unpublished null findings. The intervention heterogeneity across studies, including variations in music characteristics (tempo, volume, genre), delivery methods (headphones vs. environmental sound), and timing protocols, complicates direct comparisons and mechanistic interpretations.

### 4.6 Strengths and methodological rigor

This review represents several methodological advances over previous syntheses of music and exercise performance. The specific focus on combat sports provides unprecedented insight into a distinct athletic population with unique performance demands and cultural contexts. The inclusion of combined intervention analyses addresses a critical gap in the literature, moving beyond simple music-only effects to examine clinically relevant multimodal protocols. The comprehensive search strategy across five major databases, combined with prospective protocol registration and adherence to PRISMA 2020 guidelines, ensures systematic and unbiased evidence synthesis. The application of robust statistical methods, including random-effects meta-analysis with restricted maximum likelihood estimation and comprehensive sensitivity analyses, enhances confidence in the reliability of findings. The high methodological quality of included studies (66.7% achieving high-quality PEDro ratings) strengthens the evidence base, while the systematic application of GRADE criteria provides transparent quality assessment for clinical implementation. The detailed examination of domain-specific effects (psychological, physical, physiological) offers unprecedented granularity in understanding music's mechanisms of action in combat sports.

### 4.7 Practical implementation and ecological validity

Music intervention feasibility differs across competitive contexts and combat sport regulations. World Taekwondo, World Karate Federation, and major kickboxing federations prohibit electronic devices and auditory aids during competition bouts but permit music in designated warm-up areas. Consequently, practical protocols target pre-bout preparation (20–45 min before competition) when dopaminergic activation and arousal optimization can influence subsequent performance through residual neurobiological effects lasting 30–60 min post-exposure. Training implementation faces fewer restrictions. Music integration during moderate-intensity technical sessions, conditioning work, and skill refinement capitalizes on concurrent neurobiological effects (arousal modulation, perceived exertion reduction, enjoyment enhancement) without interfering with coach instruction or partner communication. High-intensity sparring sessions may benefit from music during rest intervals rather than active rounds to preserve attentional resources for tactical decision-making. Periodization considerations include increasing music exposure frequency during competition mesocycles (final 3–4 weeks pre-competition) to stabilize protocol familiarity and minimize novelty-related arousal perturbations. Combined interventions (particularly music + caffeine) require competition-specific trialing during training to identify optimal timing: caffeine 45–60 min pre-competition coinciding with music-enhanced warm-up 20–30 min pre-bout. Athlete autonomy in music selection is critical; coaches should facilitate playlist development reflecting individual preferences and arousal regulation needs rather than imposing standardized selections. Practical barriers include venue acoustic constraints, equipment availability (wireless headphones, portable speakers), and individual athlete preferences for silence or self-generated psychological preparation.

### 4.8 Future research priorities and methodological requirements

Future research must address methodological limitations constraining current evidence. Adequately powered trials require prospective sample size calculation; detecting small effects (*d* = 0.2) with 80% power (*α* = 0.05, two-tailed) necessitates *n* = 394 per group for parallel designs or *n* = 199 for crossover designs assuming *r* = 0.5 within-subject correlation. Prospective registration (ClinicalTrials.gov, PROSPERO) with pre-specified primary outcomes, analysis plans, and stopping rules is essential to minimize reporting bias and analytical flexibility. Grappling combat sports (judo, wrestling, Brazilian jiu-jitsu) remain underrepresented (*k* = 0 in current review), limiting generalizability across combat sport modalities with distinct energetic and technical demands. Standardized outcome batteries should employ sport-specific validated tests: taekwondo-specific agility test, frequency-speed kick test, and simulated competition protocols with temporal-technical analysis. Psychological assessment should prioritize validated instruments (PACES, feeling scale, STAI) administered at standardized time points. Mechanistic neuroimaging substudies employing functional MRI, PET dopamine imaging, or high-density EEG during music + caffeine protocols would elucidate neurobiological pathways and identify predictive biomarkers. Pharmacogenetic research investigating CYP1A2-ADORA2A-DRD2 haplotype interactions with combined intervention responses enables precision medicine implementation. Individual participant data meta-analysis consortia pooling raw data across studies permit person-level moderator modeling (baseline arousal, personality traits, genetic variants) unachievable with aggregate study-level data. All future trials should mandate open data sharing via persistent repositories (Open Science Framework, Figshare) with analysis code (R, Python) and materials (intervention protocols, assessment instruments) deposited contemporaneous with publication. Long-term investigations examining chronic adaptation patterns, tolerance development across competitive seasons, and optimal periodization strategies (loading/tapering phases) address applied questions unresolved by acute effect studies dominating current evidence.

### 4.9 Practical recommendations

Personalized implementation requires athlete-specific assessment rather than standardized protocols. Practitioners should: (1) conduct individual music preference profiling using validated instruments (Brunel Music Rating Inventory-3) to identify motivational and arousal-regulating selections; (2) match tempo to athlete's optimal arousal zone via systematic feeling scale monitoring across training sessions; (3) phenotype caffeine response through graduated dose trials (1.5, 3.0, 4.5 mg/kg) assessing performance benefits against anxiety/jitteriness side effects; (4) for female athletes, initiate with lower caffeine doses (2.0 mg/kg) given slower metabolism and adjust based on individual tolerance; (5) implement 4-week familiarization during training before competition deployment to minimize novelty-related perturbations. Population-level effect sizes (*d* = 0.19 music-only; *d* = 1.24 music + caffeine) represent central tendencies; individual responses span wide ranges (prediction interval: −1.42 to 1.80), with some athletes experiencing negligible or negative effects necessitating alternative arousal regulation strategies. The optimal evidence-based approach combines preferred music (120 BPM, 70–80 dB intensity) during warm-up with low-dose caffeine supplementation (3 mg/kg body weight) administered 30–45 min before training or competition. Individual responsiveness varies significantly based on genetic factors (CYP1A2, ADORA2A polymorphisms), musical preferences, and sport-specific demands, necessitating personalized protocols with systematic monitoring of psychological responses using validated measures (feeling scale, PACES, RPE). Athletes seeking primary psychological enhancement may benefit from music-only interventions, particularly for mood regulation and perceived exertion reduction. Practitioners should recognize that music's effects operate primarily through dopaminergic and opioidergic pathways affecting psychological rather than physiological outcomes, making these interventions most appropriate for sports emphasizing mental preparation and motivation. Regular assessment and protocol adjustment ensure sustained effectiveness while avoiding potential habituation or adverse responses.

### 4.10 Safety considerations for combined interventions

Music + caffeine protocols require safety assessment given inter-individual variability in caffeine response and potential anxiogenic effects under competitive stress. The 3 mg/kg dose employed across included combination studies (∼200–210 mg absolute dose for 70 kg athlete) remains below thresholds associated with performance decrements (∼9 mg/kg) or World Anti-Doping Agency prohibitions (urinary caffeine >15 μg/mL, achievable only at ∼9–13 mg/kg) (42). However, CYP1A2 polymorphisms create 3–40 fold inter-individual variation in caffeine metabolism (49). Athletes with AA genotype (fast metabolizers, ∼45% prevalence) exhibit rapid caffeine clearance (half-life ∼3 h) and optimal ergogenic responses at standard doses. AC and CC genotypes (slow metabolizers, ∼55% combined prevalence) demonstrate prolonged half-lives (5–7 h) with elevated adverse effect risk (anxiety, jitteriness, sleep disruption) at equivalent doses, necessitating dose reduction (1.5–2.0 mg/kg) or extended pre-exercise timing (75–90 min) (54). ADORA2A polymorphisms further modulate anxiety responses, with T/T genotype exhibiting heightened anxiogenic sensitivity under combined caffeine-stress exposure (47). Practical safety protocols include: (1) pre-competition genotyping (where accessible) or empirical dose-response phenotyping during low-stakes training; (2) systematic anxiety monitoring via State-Trait Anxiety Inventory during training trials; (3) competition-day dose individualization based on pre-competition arousal states (reduce dose if baseline arousal exceeds optimal zone); (4) 4-week minimum familiarization with consistent dose-timing protocols; (5) avoidance of novel dosing on competition day. Athletes experiencing tachycardia, tremor, or excessive anxiety during training trials should discontinue caffeine co-intervention and utilize music-only protocols. Pre-existing anxiety disorders, cardiovascular conditions, or concurrent stimulant medications represent relative contraindications requiring medical consultation before implementation.

## 5 Conclusion

This systematic review and meta-analysis demonstrate that music interventions alone produce small beneficial effects on combat sport performance through dopaminergic and opioidergic pathways that primarily influence psychological outcomes. However, the substantially superior efficacy of combined interventions, particularly music plus caffeine supplementation, reveals sophisticated neuropharmacological synergies that optimize performance enhancement through convergent effects on adenosine-dopamine systems. The considerable between-study heterogeneity reflects genuine individual and contextual variability rather than methodological inconsistencies, emphasizing the critical importance of personalized approaches considering genetic factors, musical preferences, and sport-specific demands. These findings provide practitioners with mechanistically-informed, evidence-based guidance for implementing music interventions while highlighting the substantial potential for precision medicine approaches in combat sport performance optimization.

## Data Availability

All data extraction files, analysis code, and supplementary materials are publicly available in the Open Science Framework repository at https://osf.io/tuysf/overview. Individual study data can be obtained from corresponding authors of primary studies.
