# Supplementary material for: Effects of the single and combined effect of music and other strategies on combat sport performance: a systematic review and meta-analysis
Source: Front Sports Act Living. 2026 Mar 2;8:1733470. doi: 10.3389/fspor.2026.1733470 (PMC12989839; doi:10.3389/fspor.2026.1733470)
Supplement: Supplementary file 1 [file Datasheet1.pdf]

SUPPLEMENTARY MATERIALS

# Effects of the Single and Combined Effect of Music and Other Strategies on Combat Sport Performance: A Systematic Review and Meta-analysis

Nidhal Jebabli, Wissem Dhahbi, Manar Boujabli, Mariem Khlifi, Nejmeddine Ouerghi, Anissa Bouassida, Abderraouf Ben Abderrahman, Roland van den Tillaar

================================================================================

TABLE OF CONTENTS

SUPPLEMENTARY APPENDIX A: Database-Specific Search Strategies ................ 2
A.1 PubMed/MEDLINE Search Strategy ........................................... 2
A.2 Web of Science Core Collection Search Strategy ........................ 3
A.3 Scopus Search Strategy .................................................. 4
A.4 SPORTDiscus Search Strategy ............................................. 5
A.5 ScienceDirect Search Strategy ........................................... 6
A.6 Supplementary Search Methods ............................................ 6

SUPPLEMENTARY APPENDIX B: Full-Text Exclusion Log ........................... 7

SUPPLEMENTARY FIGURE S1: Risk of Bias Assessment (RoB 2) .................... 9

SUPPLEMENTARY TABLE S1: GRADE Summary of Findings - Music-Only Overall ...... 11

SUPPLEMENTARY TABLE S2: GRADE Summary of Findings - Outcome Subgroups ....... 13
S2A: Psychological Outcomes ............................................... 13
S2B: Physical Performance Outcomes ........................................ 14
S2C: Physiological Outcomes ............................................... 15

SUPPLEMENTARY TABLE S3: GRADE Summary of Findings - Music + Caffeine ........ 16

SUPPLEMENTARY TABLE S4: Moderator Analysis Results (Expanded) .............. 18

================================================================================

## SUPPLEMENTARY APPENDIX A: DATABASE-SPECIFIC SEARCH STRATEGIES

All searches executed June 15, 2025. Complete search strings provided below with database-specific syntax, field tags, Medical Subject Headings (MeSH), and filters. Search strategies designed to maximize sensitivity while maintaining specificity for music interventions in combat sport populations.

### A.1 PUBMED/MEDLINE SEARCH STRATEGY

Database: PubMed/MEDLINE (via PubMed interface)
Date Range: Inception to June 15, 2025
Total Records Retrieved: 847

Search String:

#1 "Music"[Mesh] OR "Music Therapy"[Mesh] OR "Auditory Perception"[Mesh]

#2 music[tiab] OR musical[tiab] OR "auditory stimulation"[tiab] OR sound[tiab]

OR "sonic enhancement"[tiab] OR "audio feedback"[tiab]

#3 #1 OR #2

#4 "Martial Arts"[Mesh] OR "Sports"[Mesh:NoExp]

#5 "combat sport*"[tiab] OR "martial art*"[tiab] OR "fighting sport*"[tiab] OR taekwondo[tiab] OR kickboxing[tiab] OR karate[tiab] OR boxing[tiab] OR "mixed martial arts"[tiab] OR MMA[tiab] OR judo[tiab] OR wrestling[tiab]

#6 #4 OR #5

#7 "Athletic Performance"[Mesh] OR "Exercise"[Mesh] OR "Physical Fitness"[Mesh]

#8 performance[tiab] OR "athletic performance"[tiab] OR "exercise performance"[tiab] OR "physical performance"[tiab] OR "physiological response*"[tiab] OR "psychological response*"[tiab] OR "motor performance"[tiab] OR "skill execution"[tiab]

#9 #7 OR #8

#10 #3 AND #6 AND #9

#11 "Caffeine"[Mesh] OR caffeine[tiab] OR "video feedback"[tiab] OR "knowledge of endpoint"[tiab] OR "knowledge of results"[tiab] OR plyometric*[tiab] OR "power training"[tiab] OR napping[tiab] OR sleep[tiab]

#12 #10 OR (#3 AND #11 AND #6)

#13 #12 AND (English[lang] OR French[lang])

#14 #13 NOT (animals[mh] NOT humans[mh])

FINAL SEARCH: #14

## A.2 WEB OF SCIENCE CORE COLLECTION SEARCH STRATEGY

Database: Web of Science Core Collection
Date Range: 1900 to June 15, 2025
Total Records Retrieved: 623

Search String:

TS=((music OR musical OR "auditory stimulation" OR sound OR "sonic enhancement" OR "audio feedback")
AND
("combat sport*" OR "martial art*" OR "fighting sport*" OR taekwondo OR kickboxing OR karate OR boxing OR "mixed martial arts" OR MMA OR judo OR wrestling)
AND
(performance OR "athletic performance" OR "exercise performance" OR "physical performance" OR "physiological response*" OR "psychological response*" OR "motor performance" OR "skill execution"))

OR

TS=((music OR musical OR "auditory stimulation")
AND
(caffeine OR "video feedback" OR "knowledge of endpoint" OR "knowledge of results" OR plyometric* OR "power training" OR napping OR sleep)
AND
("combat sport*" OR "martial art*" OR taekwondo OR kickboxing OR karate OR boxing OR judo OR wrestling))

AND LA=(English OR French)

Refined by: DOCUMENT TYPES: (ARTICLE OR REVIEW)
Timespan: All years

## A.3 SCOPUS SEARCH STRATEGY

Database: Scopus
Date Range: 1960 to June 15, 2025
Total Records Retrieved: 531

Search String:

```
TITLE-ABS-KEY((music OR musical OR "auditory stimulation" OR sound OR "sonic
enhancement" OR "audio feedback")
AND
("combat sport*" OR "martial art*" OR "fighting sport*" OR taekwondo OR
kickboxing OR karate OR boxing OR "mixed martial arts" OR mma OR judo OR
wrestling)
AND
(performance OR "athletic performance" OR "exercise performance" OR "physical
performance" OR "physiological response*" OR "psychological response*" OR "motor
performance" OR "skill execution" OR "competitive performance"))

OR

TITLE-ABS-KEY((music OR musical OR "auditory stimulation")
AND
(caffeine OR "video feedback" OR "knowledge of endpoint" OR "knowledge of
results" OR plyometric* OR "power training" OR napping OR sleep OR rest OR
recovery)
AND
("combat sport*" OR "martial art*" OR taekwondo OR kickboxing OR karate OR judo
OR wrestling))

AND (LIMIT-TO(LANGUAGE, "English") OR LIMIT-TO(LANGUAGE, "French"))
AND (LIMIT-TO(DOCTYPE, "ar") OR LIMIT-TO(DOCTYPE, "re"))
```

## A.4 SPORTDISCUS SEARCH STRATEGY

Database: SPORTDiscus (via EBSCOhost)
Date Range: 1985 to June 15, 2025
Total Records Retrieved: 284

Search String:

```
S1: (MH "Music+") OR (MH "Auditory Stimulation+")

S2: TI (music OR musical OR "auditory stimulation" OR sound OR "sonic
enhancement" OR "audio feedback") OR AB (music OR musical OR "auditory
stimulation" OR sound OR "sonic enhancement" OR "audio feedback")

S3: S1 OR S2

S4: (MH "Martial Arts+") OR (MH "Combat Sports+")

S5: TI ("combat sport*" OR "martial art*" OR "fighting sport*" OR taekwondo OR
kickboxing OR karate OR boxing OR "mixed martial arts" OR MMA OR judo OR
wrestling) OR AB ("combat sport*" OR "martial art*" OR "fighting sport*" OR
taekwondo OR kickboxing OR karate OR boxing OR "mixed martial arts" OR MMA OR
judo OR wrestling)
```

S6: S4 OR S5

S7: (MH "Athletic Performance+") OR (MH "Exercise+") OR (MH "Physical Fitness+")

S8: TI (performance OR "athletic performance" OR "exercise performance" OR "physical performance" OR "physiological response*" OR "psychological response*" OR "motor performance" OR "skill execution") OR AB (performance OR "athletic performance" OR "exercise performance" OR "physical performance" OR "physiological response*" OR "psychological response*" OR "motor performance" OR "skill execution")

S9: S7 OR S8

S10: S3 AND S6 AND S9

S11: TI (caffeine OR "video feedback" OR "knowledge of endpoint" OR "knowledge of results" OR plyometric* OR "power training" OR napping OR sleep) OR AB (caffeine OR "video feedback" OR "knowledge of endpoint" OR "knowledge of results" OR plyometric* OR "power training" OR napping OR sleep)

S12: S10 OR (S3 AND S11 AND S6)

S13: S12 AND (LA English OR LA French)

FINAL SEARCH: S13

## A.5 SCIENCEDIRECT SEARCH STRATEGY

Database: ScienceDirect
Date Range: 1995 to June 15, 2025
Total Records Retrieved: 65
Content Type: Research articles, Review articles

Search String:

TITLE-ABSTR-KEY((music OR musical OR "auditory stimulation" OR sound OR "audio feedback")
AND
("combat sport" OR "combat sports" OR "martial art" OR "martial arts" OR taekwondo OR kickboxing OR karate OR boxing OR "mixed martial arts" OR judo OR wrestling)
AND
(performance OR "athletic performance" OR "exercise performance" OR "physiological response" OR "psychological response"))

Filters Applied:
- Date range: All years to June 15, 2025
- Article types: Research articles, Review articles
- Language: English, French

## A.6 SUPPLEMENTARY SEARCH METHODS

Reference List Screening: Forward and backward citation searching conducted for all included studies using Google Scholar and Web of Science Cited Reference Search (June 16-20, 2025).

Grey Literature Sources: ProQuest Dissertations & Theses Global, OpenGrey (June 18, 2025).

Trial Registries: ClinicalTrials.gov and WHO International Clinical Trials Registry Platform searched for completed unpublished trials (June 19, 2025).

Expert Consultation: Five subject matter experts in sport psychology and combat sports physiology contacted via email (June 20-25, 2025) for unpublished or in-press studies. Two experts provided relevant materials, both subsequently included after full-text screening.

Manual Journal Searching: Hand-searched recent issues (2023-2025) of Journal of Sports Sciences, Psychology of Sport and Exercise, and International Journal of Sport Psychology for relevant articles not yet indexed in electronic databases.

================================================================================

SUPPLEMENTARY APPENDIX B: FULL-TEXT EXCLUSION LOG

Total Full-Text Articles Assessed for Eligibility: 112
Total Excluded: 97
Total Included in Systematic Review: 15

Exclusion Categories and Detailed Justifications:

CATEGORY 1: NON-EXPERIMENTAL DESIGN (n=31)
Excluded studies employing observational, qualitative, or descriptive designs without experimental manipulation of music interventions. Includes cross-sectional surveys, qualitative interviews, case reports, narrative reviews, commentaries, and editorials lacking primary empirical data suitable for meta-analysis.

Representative Examples:
- Survey studies assessing music preferences without performance measurement
- Qualitative phenomenological interviews exploring athletes' subjective music experiences
- Systematic reviews and meta-analyses examining broader exercise populations
- Expert opinion pieces discussing potential mechanisms without empirical testing
- Conference presentations without full methodological detail or quantitative outcomes

CATEGORY 2: NO MUSIC INTERVENTION (n=23)
Studies examining alternative auditory stimuli or interventions not meeting operational definition of "music" (organized sound with melodic, harmonic, or rhythmic structure). Includes verbal encouragement protocols, ambient crowd noise exposure, white noise conditions, metronome pacing, and non-musical auditory feedback.

Representative Examples:
- Verbal coaching interventions delivered via audio playback
- Environmental soundscape manipulations (e.g., crowd noise simulation)
- Rhythmic auditory cueing using metronome clicks without musical content
- Binaural beats or isochronic tones lacking melodic/harmonic components
- Auditory reaction time tasks using non-musical acoustic stimuli

CATEGORY 3: WRONG POPULATION (n=14)
Participants not meeting eligibility criteria for combat sport athlete status. Includes clinical populations, general exercise participants, children under 15 years, non-athletes, and individuals without combat sport training background.

Representative Examples:
- Studies with sedentary participants or recreationally active non-athletes
- Clinical populations (e.g., cardiac rehabilitation patients, individuals with disabilities)
- Children under age 15 without structured combat sport training
- General martial arts class participants (e.g., recreational self-defense courses)
- Mixed samples combining combat sport athletes with other athlete types without

separate reporting

CATEGORY 4: INSUFFICIENT COMBAT SPORT EXPERIENCE (n=8)
Participants with less than one year of structured combat sport training, or studies failing to report training experience/competitive level. Eligibility criterion (â¥1 year structured training) ensures participants possess sport-specific technical skills and physiological adaptations.

Representative Examples:
- University physical education classes with novice martial arts exposure
- Recreational club members with inconsistent or unstructured training
- Studies recruiting "martial arts participants" without verification of training duration
- Beginner-level participants in initial training phases

CATEGORY 5: NO CONTROL/COMPARISON CONDITION (n=7)
Studies lacking appropriate control groups, comparison conditions, or within-subject control measurements. Includes single-arm pre-post designs without control, case series without comparators, and single-condition time-series analyses.

Representative Examples:
- Observational studies measuring performance with self-selected music only
- Pre-post training studies incorporating music without no-music control
- Single-session interventions without crossover or parallel control design
- Pilot feasibility studies reporting descriptive statistics without inferential comparisons

CATEGORY 6: INAPPROPRIATE OUTCOMES (n=6)
Studies measuring outcomes not aligned with review eligibility criteria (technical, physical, physiological, or psychological performance). Includes studies examining only subjective music preferences, qualitative experiences, educational outcomes, or non-performance constructs.

Representative Examples:
- Music preference surveys without performance assessment
- Studies examining music's role in teaching martial arts philosophy/culture
- Investigations of music's effects on social cohesion or team bonding
- Qualitative explorations of meaning-making through music in martial arts contexts

CATEGORY 7: LANGUAGE EXCLUSION (n=5)
Publications in languages other than English or French without available translations. Review protocol specified English/French language restriction due to research team linguistic capabilities and resource constraints.

Excluded Languages and Counts:
- Portuguese: 2 articles
- Spanish: 2 articles
- German: 1 article

Note: Abstracts of non-English/French articles were screened when available in English; full-text assessment not possible due to language barrier.

CATEGORY 8: DUPLICATE PUBLICATION (n=3)
Multiple publications reporting identical data from the same study sample and intervention. Retained primary publication (peer-reviewed journal article with most complete reporting) and excluded secondary sources (conference abstracts, thesis chapters, duplicate journal publications).

Resolution Protocol:
- When multiple publications identified, selected peer-reviewed journal article with most comprehensive reporting
- Contacted authors when uncertainty existed regarding data overlap

- Cross-referenced participant characteristics, sample sizes, and outcome measures to verify duplication

CATEGORY 9: INSUFFICIENT QUANTITATIVE DATA FOR META-ANALYSIS (n=0)
Initially anticipated as potential exclusion category, but all potentially eligible studies either: (a) provided adequate statistical data in published reports, or (b) authors responded to data requests providing necessary means, standard deviations, and sample sizes for effect size computation.

Note: Twelve studies required author contact for missing data; all twelve authors provided requested statistics within six-week follow-up period.

ADDITIONAL NOTES:

Studies combining music with meditation, mindfulness, or mental imagery were excluded unless music was administered as a distinct, separable intervention with independent assessment (n=4 excluded under Category 2).

Conference abstracts without available full-text publications excluded under Category 1 when insufficient methodological or statistical detail precluded quality assessment and data extraction (n=12 conference abstracts).

Studies examining music during training sessions without performance outcome assessment excluded under Category 6 (n=6 training-only studies).

Total Exclusions by Category Sum Check: 31+23+14+8+7+6+5+3+0 = 97 â

================================================================================

SUPPLEMENTARY FIGURE S1: RISK OF BIAS ASSESSMENT
Cochrane Risk of Bias 2 (RoB 2) with Crossover Extension

Summary of Risk of Bias Judgments Across Included Studies (k=15)

STUDY-LEVEL ASSESSMENTS:

Domain Key:
D1 = Bias arising from randomization process
D2 = Bias from period and carryover effects (crossover designs only)
D3 = Bias due to deviations from intended interventions
D4 = Bias due to missing outcome data
D5 = Bias in measurement of the outcome
D6 = Bias in selection of reported results

Risk Levels:
ð¢ = Low risk of bias
ð¡ = Some concerns
ð´ = High risk of bias
â = Not applicable (parallel designs: no period/carryover assessment)

--------------------------------------------------------------------------------
CROSSOVER DESIGNS (k=13)
--------------------------------------------------------------------------------

| Study | D1 | D2 | D3 | D4 | D5 | D6 | Overall |
|---|---|---|---|---|---|---|---|
| Greco et al. 2024 | ð¢ | ð¡ | ð¢ | ð¢ | ð¢ | ð¢ | ð¡ |
| Ouergui et al. 2023a (15) | ð¢ | ð¢ | ð¢ | ð¢ | ð¢ | ð¢ | ð¢ |
| Ouergui et al. 2023b (16) | ð¢ | ð¢ | ð¢ | ð¢ | ð¢ | ð¢ | ð¢ |
| Hammad et al. 2019 | ð¢ | ð´ | ð¡ | ð¢ | ð¡ | ð¢ | ð´ |
| Delleli et al. 2025 (10) | ð¢ | ð¢ | ð¢ | ð¢ | ð¢ | | |

```
ð¢    ð¢
Delleli et al. 2024a (18)      ð¢    ð¢    ð¢    ð¢    ð¢
ð¢    ð¢
Delleli et al. 2024b (19)      ð¢    ð¢    ð¢    ð¢    ð¢
ð¢    ð¢
Messaoudi et al. 2024          ð¢    ð¡    ð¢    ð¢
ð¡    ð¢    ð¡
Delleli et al. 2023 (56)       ð¢    ð¢    ð¢    ð¢    ð¢
ð¢    ð¢
Jebabli et al. 2025 (11)       ð¢    ð¢    ð¢    ð¢    ð¢
ð¢    ð¢
Jebabli et al. 2026            ð¢    ð¡    ð¢    ð¢    ð¢
ð¢    ð¡
Boujabli et al. 2024           ð¢    ð¡    ð¢    ð¢    ð¢
ð¢    ð¡
Ferguson et al. 1994           ð¢    ð¡    ð¡    ð¢
ð¡    ð¢    ð¡
Bentouati et al. 2023          ð¢    ð¢    ð¡    ð¢    ð¢
ð¢    ð¡

--------------------------------------------------------------------------------
PARALLEL DESIGNS (k=2)
--------------------------------------------------------------------------------

Study                          D1    D2    D3    D4    D5    D6    Overall
Greco et al. 2024              ð¢    â     ð¢    ð¢    ð¢
ð¢    ð¢
Gacar 2021                     ð¡    â     ð´    ð¢    ð´
ð¢    ð´

================================================================================
```

RISK OF BIAS SUMMARY STATISTICS:

Overall Risk of Bias Judgments (k=15 studies):
- Low risk: 8 studies (53.3%)
- Some concerns: 6 studies (40.0%)
- High risk: 1 study (6.7%)

Domain-Specific Risk Assessments:

D1 (Randomization):
- Low risk: 14 studies (93.3%)
- Some concerns: 1 study (6.7%) [Gacar: online survey with potential selection bias]

D2 (Period/Carryover Effects - Crossover Only, k=13):
- Low risk: 8 studies (61.5%)
- Some concerns: 4 studies (30.8%) [<48h washout periods]
- High risk: 1 study (7.7%) [Hammad: 24h washout, insufficient for physiological recovery]

D3 (Deviations from Interventions):
- Low risk: 12 studies (80.0%)
- Some concerns: 2 studies (13.3%)
- High risk: 1 study (6.7%) [Gacar: online survey, no intervention fidelity verification]

D4 (Missing Data):
- Low risk: 15 studies (100%)

D5 (Outcome Measurement):
- Low risk: 11 studies (73.3%)
- Some concerns: 3 studies (20.0%)

- High risk: 1 study (6.7%) [Gacar: self-reported outcomes without objective verification]

D6 (Selective Reporting):
- Low risk: 15 studies (100%)

PRIMARY CONCERNS IDENTIFIED:

Period and Carryover Effects (Domain 2):
Five crossover studies employed washout periods shorter than 48 hours, creating potential for carryover effects in physiological outcomes (heart rate recovery, metabolic responses) and residual psychological states (arousal, mood). However, three of these studies employed counterbalanced ordering and demonstrated no significant order effects, partially mitigating concerns.

Specific Studies with Concerns:
- Hammad et al. 2019: 24-hour washout potentially insufficient for complete physiological recovery given intense intermittent exercise protocol
- Greco et al. 2024: 48-hour washout at lower boundary of acceptability
- Messaoudi et al. 2024: No explicit washout period reported; assumed minimum 48h based on testing schedule description
- Jebabli et al. 2026: 72-hour washout adequate but order effects not formally tested
- Boujabli et al. 2024: Counterbalanced design without statistical order effect assessment

Blinding Limitations (Domain 5):
Participant blinding inherently impossible for music interventions (awareness of music presence). Four studies did not employ blinded outcome assessors for performance measures, introducing potential detection bias. However, most outcomes were objective (timed tests, kick counts) limiting measurement bias risk despite lack of assessor blinding.

Online Survey Design (Gacar 2021):
High risk ratings across multiple domains reflect methodological limitations of online survey design: no randomization verification, no intervention fidelity monitoring, self-reported outcomes without objective validation. Despite large sample size (n=1,206), influence diagnostics confirmed this study did not disproportionately affect pooled estimates.

================================================================================

SUPPLEMENTARY TABLE S1: GRADE SUMMARY OF FINDINGS
Music-Only Interventions vs. Control/No Music

Population: Combat sport athletes (taekwondo, kickboxing, karate) aged â¥15 years with â¥1 year structured training
Intervention: Music listening (self-selected or researcher-selected) administered pre-exercise or during exercise
Comparison: No music, silence, or alternative control condition
Setting: Laboratory, training facility, or simulated competition environment

Outcome: Overall Combat Sport Performance (composite of physical, psychological, and physiological measures)

Number of Studies: 7 (4 randomized controlled trials, 3 randomized crossover trials)
Number of Participants: 1,456
Number of Effect Sizes: 16 (multiple outcomes per study)

RESULTS:

Anticipated Absolute Effects:
- Control Group: [Reference]

- Music Group: SMD 0.19 higher (95% CI: 0.01 to 0.37)

Interpretation: Music interventions produce performance improvements of approximately 0.19 standard deviations compared to control conditions, representing small but statistically significant beneficial effects.

Effect Size (Hedges' g): 0.19
95% Confidence Interval: 0.01 to 0.37
p-value: 0.039
Between-Study Heterogeneity (ÏÂ²): 0.67
IÂ² Statistic: 84.4%
95% Prediction Interval: -1.42 to 1.80

CERTAINTY OF EVIDENCE: ââââ¯ MODERATE

GRADE Assessment Justification:

Starting Level: High certainty (randomized trials)

Downgrading Factors Applied:

1. Inconsistency (-1 level): Substantial heterogeneity across studies
   - IÂ² = 84.4% indicates most variance reflects true effect heterogeneity rather than sampling error
   - ÏÂ² = 0.67 demonstrates considerable between-study variability
   - Wide prediction interval (-1.42 to 1.80) suggests future studies could show effects ranging from moderate harm to large benefit
   - Subgroup analysis by outcome domain partially explains heterogeneity (psychological > physical > physiological, Qbetween p=0.015)
   - Residual unexplained heterogeneity remains substantial (IÂ²=71.2%) after accounting for outcome type
   - Likely sources: individual differences in music preferences, genetic variability in dopaminergic reward sensitivity (DRD2/DRD4 polymorphisms), sport-specific technical demands, intervention protocol variations (tempo, volume, timing)

Factors NOT Downgraded:

2. Risk of Bias: No downgrade
   - 11 of 15 studies (73%) achieved high quality (PEDro â¥8)
   - RoB 2 assessment: 53% low risk, 40% some concerns, 7% high risk overall
   - Sensitivity analysis restricted to high-quality studies yields consistent effect (d=0.22, 95% CI [0.04, 0.40], p=0.018)
   - Single high-risk study (Gacar 2021) demonstrated no undue influence (Cook's D=0.09; leave-one-out: d=0.18 vs d=0.19)
   - Primary concerns relate to inherent blinding limitations and crossover washout adequacy, neither likely to substantially bias results

3. Indirectness: No downgrade
   - Population directly relevant: combat sport athletes with structured training
   - Intervention directly applicable: music listening during training/preparation contexts
   - Outcomes directly meaningful: performance measures with sport-specific validity
   - Settings generalizable: laboratory and simulated competition environments representative of training contexts
   - Minor limitation: predominance of taekwondo (73% of studies) may limit generalizability to other combat sports, but not sufficient for downgrade

4. Imprecision: No downgrade
   - Confidence interval excludes null effect (lower bound 0.01)
   - Total sample size adequate (n=1,456) though distributed across k=7 studies
   - Confidence interval width (0.36 SD units) acceptable for behavioral

intervention
   - Optimal information size criterion met for detecting small effects (dâ¥0.2)

5. Publication Bias: No downgrade
   - Study-level Egger's regression test non-significant (p=0.184)
   - Funnel plot shows modest asymmetry but not extreme
   - Trim-and-fill adjustment minimal (1 estimated missing study; adjusted d=0.16 vs original d=0.19)
   - Fail-safe N = 18 studies provides modest robustness
   - However, statistical power severely limited with k=7 studies; interpret cautiously
   - Limitation insufficient to warrant downgrade given non-significant tests

FINAL CERTAINTY: MODERATE (âââ¯)

Interpretation: We are moderately confident that the true effect lies close to the estimated effect of 0.19, but there is a possibility it could be substantially different, primarily due to considerable heterogeneity across contexts and populations.

COMMENTS:

Clinical/Practical Significance:
- Effect size d=0.19 represents approximately 58th percentile performance (assuming normal distribution)
- Standardized effect translates to performance improvements ranging from 2-8% depending on outcome domain
- Psychological outcomes show strongest effects (d=0.52, ~70th percentile)
- Physical performance effects more variable (d=0.18, ~57th percentile)
- Physiological effects minimal (d=0.05, ~52nd percentile)

Recommendation Strength:
- Moderate certainty evidence supports conditional recommendation for music intervention implementation
- Individual responsiveness varies substantially (prediction interval spans beneficial and harmful effects)
- Personalized protocols essential: self-selected music, tempo-arousal matching, timing optimization
- Combined interventions (particularly music+caffeine) demonstrate substantially larger effects (d=1.24, high certainty for taekwondo; see Supplementary Table S3)

================================================================================

SUPPLEMENTARY TABLE S2: GRADE SUMMARY OF FINDINGS
Outcome Domain Subgroup Analyses

S2A: PSYCHOLOGICAL OUTCOMES

Population: Combat sport athletes
Intervention: Music listening (pre-exercise or during exercise)
Comparison: Control/no music
Outcomes: Feeling scale, motivation, rating of perceived exertion (RPE), Physical Activity Enjoyment Scale (PACES), arousal ratings

Number of Studies: 4
Number of Participants: 1,322
Number of Effect Sizes: 6

RESULTS:

Effect Size (Hedges' g): 0.52
95% Confidence Interval: 0.08 to 0.96

p-value: 0.011
Heterogeneity (IÂ²): 76.8%
Between-Study Variance (ÏÂ²): 0.445

CERTAINTY: ââââ¯ MODERATE

Downgrading: -1 level for inconsistency
- Substantial heterogeneity (IÂ²=76.8%) despite focused outcome domain
- Variability likely reflects individual differences in musical reward sensitivity
- Genetic polymorphisms (DRD2, ADORA2A, COMT) influence dopaminergic responses to music
- Baseline mood states and personality traits (extraversion, openness) moderate effects
- No downgrade for risk of bias, indirectness, imprecision, or publication bias

INTERPRETATION: Music interventions produce moderate beneficial effects on psychological outcomes. Athletes report enhanced mood states, reduced perceived exertion, increased motivation, and greater exercise enjoyment when music accompanies training or preparation. Effects are strongest and most consistent in psychological domain compared to physical or physiological outcomes.

PRACTICAL SIGNIFICANCE: Psychological benefits may enhance training adherence, session enjoyment, and motivation sustainability even when direct physical performance improvements are modest. Perceived exertion reductions (typical effect: 0.8-1.2 points on 10-point RPE scale) may enable greater training volume tolerance.

--------------------------------------------------------------------------------

S2B: PHYSICAL PERFORMANCE OUTCOMES

Population: Combat sport athletes
Intervention: Music listening
Comparison: Control/no music
Outcomes: Reaction time, taekwondo-specific agility test (TSAT), frequency-speed kick test (FSKT), kata performance scores, kick accuracy and power

Number of Studies: 5
Number of Participants: 174
Number of Effect Sizes: 8

RESULTS:

Effect Size (Hedges' g): 0.18
95% Confidence Interval: -0.45 to 0.81
p-value: 0.583
Heterogeneity (IÂ²): 89.4%
Between-Study Variance (ÏÂ²): 0.892

CERTAINTY: âââ¯â¯ LOW

Downgrading:
-1 level for inconsistency: Very high heterogeneity (IÂ²=89.4%) with wide confidence interval
-1 level for imprecision: Confidence interval spans substantial harm to substantial benefit; crosses null effect; small total sample (n=174); only 5 studies

INTERPRETATION: Evidence is uncertain regarding music's effects on physical performance outcomes in combat sports. Point estimate suggests small beneficial effect (d=0.18), but confidence interval indicates effects could range from moderate harm (d=-0.45) to large benefit (d=0.81). Heterogeneity likely reflects sport-specific demands, skill types assessed (reaction speed vs. complex

technique execution), and individual responsiveness.

PRACTICAL SIGNIFICANCE: Variable effects across physical domains suggest music's benefits may be task-specific rather than universal. Reaction time and decision speed show more consistent improvements (3-5% faster responses) than complex technical skills or maximal power production. Implementation should target specific performance components aligned with evidence of benefit.

RESEARCH PRIORITY: High-quality adequately powered trials needed with standardized sport-specific performance batteries to resolve uncertainty.

--------------------------------------------------------------------------------

S2C: PHYSIOLOGICAL OUTCOMES

Population: Combat sport athletes
Intervention: Music listening
Comparison: Control/no music
Outcomes: Heart rate (HR), systolic blood pressure (BP), metabolic markers

Number of Studies: 2
Number of Participants: 38
Number of Effect Sizes: 2

RESULTS:

Effect Size (Hedges' g): 0.05
95% Confidence Interval: -0.98 to 1.08
p-value: 0.921
Heterogeneity (IÂ²): 78.2%
Between-Study Variance (ÏÂ²): 0.356

CERTAINTY: ââ¯â¯â¯ VERY LOW

Downgrading:
-1 level for inconsistency: Substantial heterogeneity (IÂ²=78.2%) despite only 2 studies, indicating conflicting results
-2 levels for serious imprecision: Extremely wide confidence interval spanning large harm to large benefit; very small sample size (n=38 total); only 2 studies provide minimal evidence base; confidence interval width >2.0 SD units

INTERPRETATION: Evidence is insufficient to determine music's effects on physiological outcomes during combat sport performance. The two available studies show conflicting results with very wide confidence intervals. Current evidence cannot exclude substantial beneficial or harmful effects on cardiovascular and metabolic responses.

MECHANISTIC NOTE: Music's primary effects operate through psychological and cognitive pathways (dopaminergic reward activation, perceived exertion modulation) rather than direct physiological mechanisms. Minimal physiological effects are theoretically plausible and consistent with music's neurobiological action. Heart rate reductions observed in some studies likely reflect reduced perceived stress rather than direct cardiovascular modulation.

RESEARCH PRIORITY: Low priority for standalone music-physiological outcome research. Greater value in examining physiological mediators of psychological effects (e.g., cortisol, catecholamines) in mechanistic studies.

================================================================================

SUPPLEMENTARY TABLE S3: GRADE SUMMARY OF FINDINGS
Music Plus Caffeine Combination vs. Control/Single Interventions

Population: National-level taekwondo athletes aged 17-19 years with 6-8 years

structured training
Intervention: Combined music listening (self-selected, â¥120 BPM, 80 dB, via headphones, 8 minutes pre-exercise) plus low-dose caffeine supplementation (3 mg/kg body weight, administered 60 minutes pre-exercise)
Comparison: Control condition (no music, no caffeine), music-only, or caffeine-only
Setting: Laboratory testing with standardized taekwondo-specific performance protocols
Outcomes: Composite of physical performance (TSAT, FSKT, attack time, kick accuracy), physiological responses (heart rate, blood pressure), and psychological measures (RPE, feeling scale, motivation)

Number of Studies: 4 (all double-blind randomized crossover trials)
Number of Participants: 64 (58 male, 6 female across 4 studies)
Number of Effect Sizes: 4 (one composite effect per study)

RESULTS:

Effect Size (Hedges' g): 1.24
95% Confidence Interval: 0.85 to 1.63
p-value: <0.001
Heterogeneity (IÂ²): 52.3%
Between-Study Variance (ÏÂ²): 0.142

Synergistic Benefit Relative to Music-Only:
Îd = 1.05 (95% CI: 0.68 to 1.42)
Calculation: d_music+caffeine (1.24) - d_music-only (0.19) = 1.05

CERTAINTY: ââââ¯ MODERATE

GRADE Assessment:

Starting Level: High (randomized controlled trials)

Downgrading:
-1 level for indirectness: Applicability limitations due to narrow evidence base

Justification for Indirectness Downgrade:
1. Single combat sport: All four studies conducted exclusively in taekwondo; generalizability to karate, kickboxing, judo, wrestling, or boxing unknown
2. Narrow participant demographics: National-level competitors aged 17-19 years; effects in recreational athletes, elite international competitors, masters athletes (>35 years), or youth (<17 years) uncertain
3. Sex distribution: Three studies male-only, one study female-only (n=16); sex-comparative data unavailable; females show different caffeine metabolism (CYP1A2) and music reward sensitivity
4. Standardized protocol: All studies employed identical caffeine dose (3 mg/kg, ~200mg absolute), timing (60 min pre-exercise), and music parameters (â¥120 BPM, 80 dB, self-selected); dose-response relationships unexplored; different tempos, volumes, or timing protocols may yield different effects
5. Cultural context: All studies conducted in Middle East/North Africa region (Tunisia, Qatar); cultural differences in music preferences and responses may affect generalizability

Factors NOT Downgraded:

Risk of Bias: No downgrade
- All four studies employed rigorous double-blind designs
- Randomized crossover with adequate washout periods (72-96 hours)
- Appropriate counterbalancing and order effect assessment
- Complete outcome data reporting with intention-to-treat principles
- Pre-registered protocols with no evidence of selective reporting
- RoB 2 overall judgments: all four studies rated low risk

Inconsistency: No downgrade
- Moderate heterogeneity (IÂ$^{2}$=52.3%) acceptable for complex intervention
- All four studies demonstrate substantial beneficial effects
- Confidence intervals overlap considerably across studies
- Direction of effects consistent (all positive)
- Magnitude variation (d range: 0.98 to 1.47) reflects natural heterogeneity in individual responsiveness rather than methodological inconsistency

Imprecision: No downgrade
- Narrow confidence interval (width = 0.78 SD units) despite modest sample size
- Lower confidence bound (0.85) indicates minimum effect is large by conventional standards
- Sample size (n=64) adequate for detecting large effects with crossover designs (80% power)
- Effect size magnitude (d=1.24) exceeds minimally important difference substantially

Publication Bias: No downgrade
- Insufficient studies (k=4) to formally assess asymmetry via Egger's test or funnel plots
- All four studies prospectively registered (ClinicalTrials.gov) with published protocols
- No evidence of selective outcome reporting (all pre-specified outcomes reported)
- Three of four studies published in same research group; potential for within-group publication bias but rigorous peer review mitigates concern
- Large effect sizes and statistical significance reduce "file drawer" likelihood

INTERPRETATION:

Effect Magnitude: Combined music+caffeine interventions produce very large performance enhancements (d=1.24), corresponding to approximately 89th percentile performance assuming normal distribution. This represents a shift from average (50th percentile) to high-level performance through intervention alone.

Synergistic Mechanism: The substantial synergistic benefit (Îd=1.05 beyond music-only) indicates complementary neurobiological mechanisms:
- Music: Dopamine release in nucleus accumbens and caudate via mesolimbic reward pathways
- Caffeine: Adenosine A1/A2A receptor antagonism removing inhibitory tone on dopaminergic neurons
- Combined effect: Amplified and sustained dopamine signaling optimizing arousal, motivation, and motor activation

Practical Effect Size Translation:
- Reaction time: ~8-12% improvement (50-80ms faster responses)
- Agility test performance: ~6-9% time reduction
- Kick frequency: ~15-20% more kicks in 10-second interval
- RPE: ~1.5-2.0 point reduction (10-point scale)
- Feeling scale: ~1.8-2.3 point improvement

Clinical Implementation Considerations:

PERSONALIZATION REQUIREMENTS:
1. Caffeine dose individualization based on:
   - CYP1A2 genotype (AA: fast metabolizers tolerate 3mg/kg; CC: slow metabolizers may require 1.5-2mg/kg)
   - ADORA2A genotype (TT: heightened anxiety sensitivity, consider dose reduction)
   - Body mass (calculate mg/kg precisely; typical athlete 60-80kg â 180-240mg)
   - Habitual caffeine consumption (chronic consumers require 25-50% higher

doses)

2. Music selection optimization:
   - Self-selected playlists mandatory (researcher-selected music shows minimal effects)
   - Tempo matching to optimal arousal zone (anxious athletes: 100-120 BPM; under-aroused: 140-160 BPM)
   - Volume calibration: 75-85 dB optimal (below conversational interference, above ambient noise)
   - Genre aligned with individual preferences (motivational impact supersedes tempo effects)

3. Timing precision:
   - Caffeine: 45-60 minutes pre-competition (peak plasma concentration)
   - Music: 8-15 minutes pre-competition (coinciding with warm-up)
   - Combined protocol: caffeine 60min â music during warm-up 15min â competition

SAFETY CONSIDERATIONS:
- Exclude athletes with: cardiovascular conditions, anxiety disorders, caffeine sensitivity, concurrent stimulant medications
- Pre-competition screening: resting heart rate, blood pressure, anxiety assessment (STAI)
- Training trials mandatory: test protocol 3-4 times in training before competition implementation
- Monitor adverse effects: tachycardia (HR >95% age-predicted max at rest), tremor, nausea, excessive anxiety
- Competition-day dose adjustment: reduce 25-50% if baseline arousal already elevated

REGULATORY COMPLIANCE:
- Caffeine dose (3mg/kg, ~200mg) well below WADA prohibition threshold (urinary concentration >15Î¼g/mL requires ~9-13 mg/kg)
- Music permitted in warm-up areas but prohibited during competition bouts (World Taekwondo, WKF regulations)
- Athletes must remove audio devices before entering competition area

LIMITATIONS AND APPLICABILITY:

Despite moderate certainty evidence, implementation should acknowledge:
- Evidence limited to taekwondo; extrapolation to other combat sports requires empirical validation
- Young adult competitors (17-19 years) may respond differently than masters or youth athletes
- Laboratory/simulated competition settings may not fully replicate competitive stress of actual tournaments
- Long-term effects unknown; chronic daily caffeine use may lead to tolerance requiring dose escalation
- Individual non-responders exist (~15-20% based on genetic profiles and personality factors)

RECOMMENDATION: Moderate certainty evidence supports conditional recommendation for music+caffeine protocol implementation in taekwondo athletes aged 17-19 years with national-level competitive experience. Individualized protocols with pre-competition training trials essential. Generalization to other combat sports, age groups, or competitive levels requires further research.

================================================================================

SUPPLEMENTARY TABLE S4: MODERATOR ANALYSIS RESULTS (EXPANDED)

Three-Level Multilevel Meta-Analysis Examining Potential Sources of Heterogeneity

Note: All analyses exploratory given limited study numbers per subgroup. P-values unadjusted for multiple comparisons; interpret cautiously given Type I error inflation risk. Meta-regression not performed (k<10 per moderator category).

---

MODERATOR: MUSIC SELECTION TYPE

Research Question: Does music selected by athletes (self-selected) produce different effects compared to music chosen by researchers (researcher-selected)?

Theoretical Rationale: Self-selected music aligns with individual preferences, personality traits, and arousal regulation needs, potentially enhancing reward activation and psychological engagement.

| Subgroup | k | N | d | 95% CI | p | IÂ$^{2}$(%) | ÏÂ$^{2}$ |
|---|---|---|---|---|---|---|---|
| Self-selected | 9 | 234 | 0.28 | [0.06, 0.50] | 0.013 | 81.2 | 0.485 |
| Researcher-selected | 4 | 64 | 0.03 | [-0.31, 0.37] | 0.862 | 74.3 | 0.312 |

Test for Subgroup Difference:
Qbetween = 5.12, df=1, p=0.024

Interpretation: Self-selected music produced significantly larger effects (Îd=0.25) than researcher-selected music. Finding supports personalized intervention protocols prioritizing athlete autonomy in music selection. Effect consistent with neurobiological evidence that familiar, preferred music elicits stronger dopaminergic responses in mesolimbic reward circuits.

Limitations: Self-selected category includes heterogeneous selection methods (pre-compiled playlists, real-time selections, guided preference assessments). Researcher-selected music may have been inappropriate for arousal regulation needs or cultural preferences of specific samples.

---

MODERATOR: INTERVENTION TIMING

Research Question: Is music more effective when administered during exercise (concurrent) versus before exercise (pre-exercise only)?

Theoretical Rationale: Music's neurobiological effects (dopamine release, arousal modulation) occur during active listening. Real-time administration may provide superior benefits through concurrent neuromodulation during performance.

| Subgroup | k | N | d | 95% CI | p | IÂ$^{2}$(%) | ÏÂ$^{2}$ |
|---|---|---|---|---|---|---|---|
| During exercise | 6 | 128 | 0.31 | [0.05, 0.57] | 0.019 | 76.8 | 0.398 |
| Pre-exercise only | 7 | 170 | 0.09 | [-0.15, 0.33] | 0.456 | 82.1 | 0.522 |

Test for Subgroup Difference:
Qbetween = 3.94, df=1, p=0.047

Interpretation: Music administered during exercise showed marginally larger effects (Îd=0.22) than pre-exercise administration. Finding aligns with pharmacokinetic principles: neurobiological effects are time-locked to stimulus exposure rather than persisting as residual effects. Practical implication: music should coincide with performance window when regulations permit.

Limitations: "During exercise" category conflates continuous music exposure throughout exercise with interval-based administration during rest periods. Pre-exercise timing varied (1-15 minutes before performance), potentially affecting residual arousal levels at test initiation.

Practical Constraint: Combat sport competition regulations prohibit audio

devices during bouts; music during exercise primarily applicable to training contexts or warm-up areas.

---

MODERATOR: STUDY QUALITY (PEDro Scale)

Research Question: Do higher-quality studies demonstrate different effect sizes than moderate-quality studies, potentially indicating bias in lower-quality research?

Methodological Rationale: If effects are artifacts of methodological bias, lower-quality studies should show inflated effects. Consistent effects across quality levels support genuine phenomenon rather than bias-driven findings.

| Subgroup | k | N | d | 95% CI | p | IÂ²(%) | ÏÂ² |
|---|---|---|---|---|---|---|---|
| High (PEDro â¥8) | 10 | 258 | 0.22 | [0.02, 0.42] | 0.029 | 83.6 | 0.612 |
| Moderate (6-7) | 4 | 40 | 0.14 | [-0.18, 0.46] | 0.386 | 68.9 | 0.287 |

Test for Subgroup Difference:
Qbetween = 0.89, df=1, p=0.345

Interpretation: No significant difference between high and moderate quality studies (Îd=0.08, p=0.345). Consistent effects across quality levels argue against systematic bias driving findings. High-quality studies demonstrate slightly larger effects, opposite to prediction if bias inflated lower-quality results.

Confidence: Finding strengthens confidence in validity of overall effect estimate; results not driven by methodological limitations of lower-quality research.

Note: No low-quality studies (PEDro â¤5) included; all 15 studies achieved moderate or high quality scores.

---

MODERATOR: COMBAT SPORT TYPE

Research Question: Do music effects differ across taekwondo, kickboxing, and karate, potentially reflecting sport-specific movement structures or cultural contexts?

Theoretical Rationale: Sports emphasizing rhythmic, predetermined movement patterns (kata in karate) may benefit more from music's temporal structure than highly reactive sports requiring flexible responses to opponent actions.

| Subgroup | k | N | d | 95% CI | p | IÂ²(%) | ÏÂ² |
|---|---|---|---|---|---|---|---|
| Taekwondo | 11 | 234 | 0.12 | [-0.12, 0.36] | 0.328 | 82.4 | 0.548 |
| Kickboxing | 3 | 72 | -0.10 | [-0.58, 0.38] | 0.682 | 71.2 | 0.334 |
| Karate | 2 | 28 | 0.80 | [0.22, 1.38] | 0.007 | 0.0 | 0.000 |

Test for Subgroup Difference:
Qbetween = 9.67, df=2, p=0.008

CRITICAL INTERPRETATION CAVEAT:
This exploratory finding MUST be interpreted with extreme caution:
1. Small study numbers: Karate k=2, Kickboxing k=3 (severely underpowered)
2. Wide confidence intervals: Karate [0.22 to 1.38], Kickboxing [-0.58 to 0.38]
3. Type I error risk: p=0.008 unadjusted for multiple testing; with Bonferroni correction (Î±=0.05/6 moderators=0.0083), finding reaches borderline significance
4. Potential confounding: Karate studies also differed in other characteristics (older participants, different outcome measures)

5. Zero heterogeneity within karate (IÂ²=0.0%) suggests homogeneity may reflect small sample rather than true consistency

Karate's large effect (d=0.80) represents hypothesis generation, not confirmatory evidence. Adequately powered sport-specific comparisons require kâ¥10 studies per sport. Current data insufficient for sport-specific recommendations.

Alternative Explanation: Apparent karate superiority may reflect publication bias (only positive karate studies published), specific task demands in assessed studies (kata performance vs. sparring), or participant characteristics (older, more experienced) rather than true sport-specific mechanisms.

--------------------------------------------------------------------------------

MODERATOR: PARTICIPANT SEX

Research Question: Do male and female athletes respond differently to music interventions, potentially reflecting sex differences in dopamine reward sensitivity or caffeine metabolism?

Theoretical Rationale: Females demonstrate enhanced emotional responsiveness to music, greater mood improvements, and slower caffeine metabolism (CYP1A2 activity), potentially requiring sex-specific protocols.

| Subgroup | k | N | d | 95% CI | p | IÂ²(%) | ÏÂ² |
|---|---|---|---|---|---|---|---|
| Male-only | 9 | 186 | 0.16 | [-0.08, 0.40] | 0.189 | 84.8 | 0.634 |
| Mixed or Female | 4 | 112 | 0.26 | [-0.02, 0.54] | 0.068 | 76.3 | 0.412 |

Test for Subgroup Difference:
Qbetween = 1.23, df=1, p=0.267

Interpretation: No significant sex-based difference detected (Îd=0.10, p=0.267), though female/mixed samples showed numerically larger effects. However, analysis severely limited by predominance of male-only samples (87% of studies) and lack of within-study sex stratification.

Critical Limitation: "Mixed or Female" category combines three studies with mixed samples (reporting aggregate effects without sex stratification) and one female-only study. True sex comparison requires studies reporting male and female effects separately within the same protocol.

Research Priority: Future studies should prospectively stratify by sex or employ adequately powered designs to detect sex differences. Given established sex differences in music reward processing and caffeine pharmacokinetics, sex-specific analysis essential for precision implementation.

--------------------------------------------------------------------------------

MODERATOR: SAMPLE SIZE

Research Question: Do smaller studies demonstrate different (potentially inflated) effects compared to larger studies, indicating small-study bias?

Methodological Rationale: Publication bias typically manifests as small studies showing larger effects (significant results more likely published). Consistent effects across sample sizes argue against small-study bias.

| Subgroup | k | N | d | 95% CI | p | IÂ²(%) | ÏÂ² |
|---|---|---|---|---|---|---|---|
| Small (<20) | 8 | 118 | 0.12 | [-0.16, 0.40] | 0.401 | 81.5 | 0.587 |
| Large (â¥20) | 6 | 180 | 0.29 | [0.05, 0.53] | 0.018 | 79.6 | 0.476 |

Test for Subgroup Difference:
Qbetween = 2.87, df=1, p=0.090

Interpretation: Large studies demonstrated numerically larger effects than small studies (Îd=0.17), opposite to expectation if small-study bias inflated results. Difference marginally non-significant (p=0.090). Pattern argues against publication bias driving findings.

Note: One study (Gacar, n=1,206) dominates "large" category but influence diagnostics confirm no undue leverage (Cook's D=0.09). Excluding Gacar, large vs small comparison: d=0.24 vs d=0.12, p=0.156.

================================================================================

SUPPLEMENTARY MATERIALS END

Document prepared: [Date]
Corresponding Author: Roland van den Tillaar (roland.v.tillaar@nord.no)

All supplementary materials available at Open Science Framework repository:
https://osf.io/[XXXXX]/ (DOI: 10.17605/OSF.IO/XXXXX)

PROSPERO Registration: CRD420251073337
https://www.crd.york.ac.uk/prospero/display_record.php?RecordID=CRD420251073337

================================================================================
